# Supplementary material for: Fine-scale population structure and demographic history of British Pakistanis
Source: Nat Commun. 2021 Dec 10;12:7189. doi: 10.1038/s41467-021-27394-2 (PMC8664933; doi:10.1038/s41467-021-27394-2)
Supplement: Supplementary file 1 — Supplementary Information [file 41467_2021_27394_MOESM1_ESM.pdf]

Supplementary Information for Fine-scale population structure and demographic history of British Pakistanis

Supplementary Notes

Supplementary Note 1

Genetic diversity of Bradford Pakistanis in a worldwide context

We first investigated the genetic relationships between the Bradford Pakistanis and other worldwide populations from publicly available datasets (HGDP, Human Origins and 1000 Genomes). Principal component analysis (PCA) [1,2] showed that Pakistanis from Bradford cluster with other South Asians, and lie between other Pakistani and Indian populations (Supplementary Figure 3a,b,d). Focusing on Pakistani groups, the Bradford Pakistanis lie between the Sindhi, Pathan and Burusho (Supplementary Figure 3b,c), as expected given they are predominantly from Punjab and Kashmir (Supplementary Figure 1). This conclusion was unchanged whether projecting all BiB Pakistanis onto the HGDP samples (Supplementary Figure 3c) or including a small subset of 25 BiB samples with the HGDP samples in the PCA (Supplementary Figure 3d).

Identification of ancestral components using ADMIXTURE [2] (Supplementary Figure 4a) showed that Bradford Pakistanis have a similar genetic profile to other South Asian populations, and display little variation in their ancestral components in this broad context. The majority of the BiB samples are most similar to the Sindhi, out of the reference populations included. Only the BiB Pathans stand out; they have a higher fraction of the pink and blue components seen in Europeans, as seen in the HGDP Pathans. This is consistent with the fact that they represent a distinct ethnic group from Punjabis and Kashmiris, speaking a language from a different family (Pashto from the Iranian family, as opposed to Punjabi from the Indo-Aryan family).

These results were confirmed comparing the Bradford Pakistanis subgroups to both modern and ancient genomes from different archaeological times and areas using *f3* statistics (Supplementary Figure 4b, Supplementary Data 4). All subgroups show a similar genetic sharing with ancient samples spanning from the Bronze Age to historical time (Supplementary Figure 4b). The Pathan show higher genetic affinity to Bronze Age steppe individuals, consistent with their geographical origin in west Pakistan and the evidence of migrations during the Bronze Age from Central Asian steppe into South Asia [3,4]. Computing outgroup *f3* statistics[5] using modern populations confirmed genetic affinity between the BiB Pakistanis and the HGDP Pathans, 1000 Genomes Punjabis, northern (Uttar Pradesh Brahmins) and western (Kashimiri Pandit) Indian populations, and Central Asia populations, consistent with previous studies that have demonstrated the ANI component in modern

Pakistanis [3,6]. The results were very similar between all the self-reported BiB sub-groups (Supplementary Data 4), so we combined them in Supplementary Figure 4c.

We modelled the Bradford subgroups as a mixture of ANI and ASI components using qpgraph as shown in [3]. Indeed, we confirmed that the ANI/ASI model fits well for the Bradford subgroups and they all have between 61% and 80% ANI ancestry, similar to previous estimates of Pakistani and northwest Indian populations [3,6,7] Pathan has the highest proportion of ANI ancestry whereas Qasabi has the lowest (Supplementary Figure 19, Supplementary Data 15). The largest deviation between theoretical and empirical *f*-statistics was |Z-score|= 2.9, suggesting a good fit of the model considering the vast number of *f*-statistics analysed.

We next investigated whether the self-reported subgroups who claim to have recent Arabic ancestry had a different pattern of genetic sharing with Middle Eastern populations compared to the other subgroups. We did not find statistically significant differences between groups using outgroup *f3* or *f4* statistics (Supplementary Figure 4d, Supplementary Data 5).

We also saw no differences in the distribution of Y chromosome haplogroups between the biraderi that report Arabic ancestry and the other subgroups. Eighty-nine percent of individuals belong to the IJ* Y haplogroups, which are prevalent in modern Pakistan and Western/Central Asia [8,9], while the rest have other haplogroups that are also present in Central and South Asia (Supplementary Data 6)[10,11]. We used aYChr-DB [12] to look for reported Y-chromosome haplogroups in ancient samples. The IJ*, G* and P* haplogroups have been found in West and Central Asia individuals from the Neolithic period onwards, including Iranian farmers and one sample from the Central Steppe, two of the major ancestral contributions to modern-day South Asians [3]. More Y chromosome SNPs would be required to give better resolution of the haplogroups. Similarly, the mothers have mitochondrial haplogroups that are common in Central and South Asia [13,14,15,16,17] (Supplementary Data 7).

Robustness of clustering approach using fineSTRUCTURE

To confirm our clustering approach using fineSTRUCTURE was not biased by a failure to remove relatives, we ran fineSTRUCTURE using a downsampling approach. We randomly downsampled to 60% of each cluster from Figure 2a four times and ran fineSTRUCTURE for each downsampled dataset. As shown in Supplementary Figure 20, our clustering approach was robust even across downsampled replicates. Although the topology of the tree changed somewhat (as expected since the tree is not rooted in the hierarchical clustering), all homogeneous groups were recapitulated. This suggests that the structure seen in Figure 2a was unlikely to be biased by the accidental inclusion of relatives.

Quality control of IBD calls and sensitivity analyses for IBD-based inference

When we plotted unfiltered IBD calls along the genome, we noticed that a suspiciously high number of IBD calls were being made in particular regions, and that these regions were enriched for gaps or overlapping centromeric regions, suggesting that these were artefacts.

For the IBDNe and IBD score figures in the main text, we thus excluded IBD segments overlapping these regions: chr1:120-152Mb, chr2:88-102Mb; chr9:38-72Mb; chr10:17-19Mb and chr11:0-3Mb. We furthermore excluded a small number of outlier IBD segments longer than 23cM that were seen with identical coordinates in more than ten pairs of unrelated (PropIBD<0.084) individuals, and some IBD segments >25cM partially overlapping the centromeric region of chr15, which were seen with identical coordinates in more than five pairs of unrelated individuals. These regions seemed likely to be spurious since they were seen across several 1000 Genomes populations and not just within the Bradford Pakistanis. Finally, we excluded IBD segments overlapping the HLA region and centromeres.

To confirm the robustness of our demographic inferences, we ran IBDNe and computed IBD scores using different filtering strategies. Similar results were seen for IBDNe when we used unfiltered rather than filtered IBD calls (Supplementary Figure 11a) and when we used GERMLINE rather than IBDseq IBD calls (Supplementary Figure 11b). The overall higher N_e estimates using GERMLINE compared to IBDSeq may be due to phasing errors; as previously reported, estimation using IBDseq is probably more accurate as it does not require phased data and, thus, is less prone to switch errors [18]. Furthermore, we obtained similar N_e estimates when comparing the (self-reported) Pathan and Gujjar subgroups in our dataset with high coverage whole-genome data from a previous publication[19]. This confirms that our IBDNe estimation is not biased by using SNP-genotype data (Supplementary Figure 11c). IBDNe results were also consistent when individuals with high levels of homozygosity (F_{ROH}>2%) were excluded (Supplementary Figure 10a). We confirmed that the slight variation in N_e trajectories was probably due to the smaller sample size rather than the level of autozygosity. To do so, we ran IBDNe randomly resampling 10 times individuals from the homogenous groups to match the sample size of low autogosity samples (Supplementary Figure 10a). We also obtained similar N_e estimations when considering the fineSTRUCTURE clusters as groups and when considering the self-declared subgroups (Supplementary Figure 10b-c). Finally, overall lower N_e was also seen when all BiB Pakistanis were compared to the BiB white British (Supplementary Figure 10d).

Similar IBD scores were seen when using the same length filtering as Nakatsuka *et al.* [20] (Supplementary Figure 12). They had used IBD segments >30cM to define and exclude possible close relatives (on top of a filter based on PLINK's $\widehat{\Pi}$), and then considered the total length of segments between 3 and 20cM. The appropriateness of these cutoffs depends on the patterns of IBD sharing within a population of interest, as well as the density of SNPs being used to call IBD segments. We found that in the BiB Pakistanis, a nontrivial fraction of individuals who were estimated to be unrelated by KING had an IBD segment between 20 and 30cM (Supplementary Figure 18), and due to the sparsity of SNPs on the CoreExome chip, we suspected IBD segments <5cM were not reliably estimated. Hence, we selected slightly different filters when preparing Figure 4 as described in the Methods section. Consistent results were retrieved when applying the same IBD cut-offs used in Nakatsuka *et al.* (Supplementary Figure 12a and c) and when focusing on the fineSTRUCTURE clusters with our approach (Supplementary Figure 12b). Consistent results were also obtained when filtering IBD calls in different ways (Supplementary Figure 12d) and when calculating the scores using IBDseq IBD calls (Supplementary Figure 12e).

Assessing accuracy of NeON divergence time estimates

To confirm that our divergence time inferences with NeON[21] were robust, we performed a series of forward simulations under different demographic scenarios using simuPOP v.1.9 [22]. We considered a model in which an ancestral population (N_A) split into two populations N_1 and N_2. We assumed the N_A=11,000, N_1=5,000 and N_2=6,000, respectively. N_1 and N_2 sizes were derived from the long-term N_e for Bains/Rajput-B and Pathan estimated with NeON:

$$(1)\ N_e \approx 1/(4c)\ *\ [(1/r^2) - 2]\ ,$$

where c is the distance between genetic markers in Morgans [23] and r^2 is the linkage disequilibrium for each pair of markers. We implemented three possible demographic scenarios in the simulations (Supplementary Figure 21). Firstly, we modelled a scenario in which N_A splits into two populations N_1 and N_2 with a constant size through time (scenario 1). Secondly, to simulate endogamy, we assumed that N_1 and N_2 would both decrease to 40% between the split and present day (scenario 2). Thirdly, we assumed that N_1 went through a progressive size reduction whereas N_2 stayed constant through time (scenario 3). Finally, we assumed an extreme scenario with N_1 and N_2 that would both decrease to 10% between the split and present day (scenario 4). We simulated 20,000 independent loci in 22 chromosomes and considered a SNP mutation rate of 2.5×10^{-8}. We computed the Weir and Cockerham F_{ST} between the simulated populations sampling at n generations after the split (1, 10, 20, 30, 40, up to 200). We then estimated the real divergence time with the NeON package using the estimated F_{ST} values according to the following formula:

$$(2)\ T_{DIV} = ln\,(1 - F_{ST})\ /\ ln\,(1 - 1/2N_e)$$

We computed mean and standard deviation across 10 replicates for each scenario for each generation sampled and compared the estimated versus simulated divergence times. Under all four scenarios tested, the method correctly infers the simulated divergence time (Supplementary Figure 21).

Inferring the degree of parental relatedness

Using all ten classes, we found that the accuracy to differentiate between these classes of parental relationship was 47% (standard deviation, SD, 0.56%). However, this modest accuracy was driven by the relatively lower sensitivity to distinguish similar types of consanguineous unions; the distributions of the statistics overlap considerably when there is a similar number of meioses separating the parents of an individual (e.g for second cousin and first cousin once removed; for avuncular or multiple generations of first cousin marriages). When grouping together similar classes and considering only three groups [i.e. 1) first cousins and closer, 2) first cousins once removed and second cousins, and 3) unrelated (more distant than second cousins)], the accuracy improved to 92.0% (SD 0.35%). When combining simulated offspring from all nine consanguineous relationships and comparing them to offspring of unrelated parents, the accuracy improved substantially to 97.8% (SD 0.57%). The confusion matrix from an example of testing the neural net classifier on simulated data is shown in Supplementary Data 16, and the positive predictive values (individuals correctly inferred in category/total inferred individuals in category) and sensitivity

(individuals correctly inferred in category/total true individuals in category) in Supplementary Data 17.

We found that the results were identical when using sex-specific recombination maps [24] and when modelling gene conversions at a rate 5.6×10^{-6} /bp/generation [25] and genotyping error at a rate of 0.001/bp/generation. The average relative importance (as defined in [26]) of each variable varied substantially across 50 sets of simulated training data but, when averaged across those 50 sets, was similar between variables. We also obtained very similar results when training the model on data simulated from a European cohort, suggesting that endogamy in the Pakistani population has minimal effect on our inference. Indeed, Supplementary Figure 15 shows that the effect of endogamy is small for ROH of length >10cM and effective population size ≥10k.

Finally, using the intersection of Core Exome and GSA data from seventy-four Pakistani trios, we found that the degree of consanguinity inferred for the child with our method was largely consistent with the KING PropIBD estimates for relatedness between the parents (Supplementary Figure 14e).

Supplementary Figures

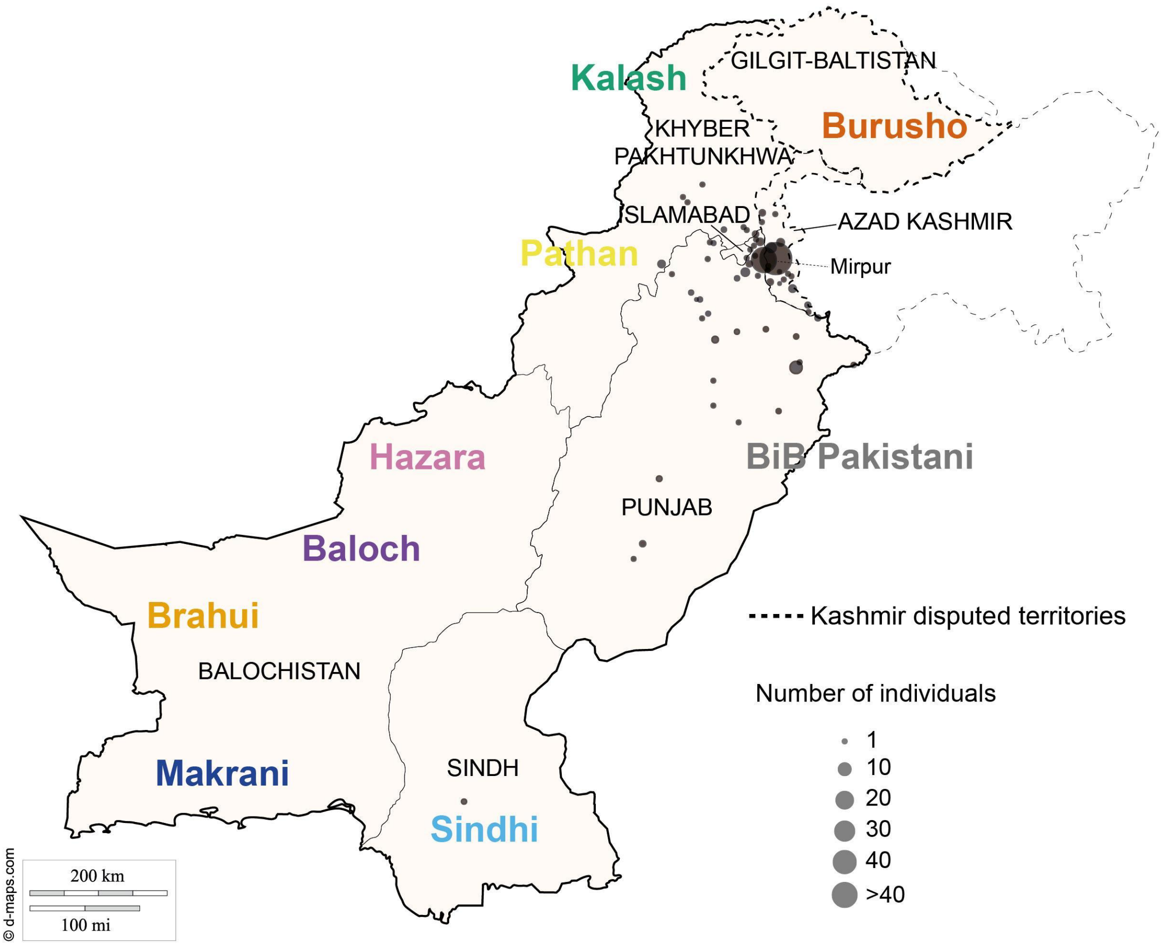

Supplementary Figure 1: Map of Pakistan indicating the approximate origin of each of the Human Genome Diversity Project (HGDP) populations in the coloured text, as well as the self-reported geographic origin location of the BiB Pakistanis (see Methods). The size of the circles is proportional to the number of individuals reporting that they originate from that location. Most of the BiB Pakistani are from Mirpur in Azad Kashmir and northern Punjab. Note that although the Pathan are concentrated in the northwest of the country, many Pathan also live in other parts of Pakistan including Punjab, Sindh and Kashmir. Kashmir disputed territories are indicated with dashed contouring on the map. Pakistan provinces are indicated in black capital letters on the map. The map has been modified from https://d-maps.com/carte.php?num_car=5567&lang=en

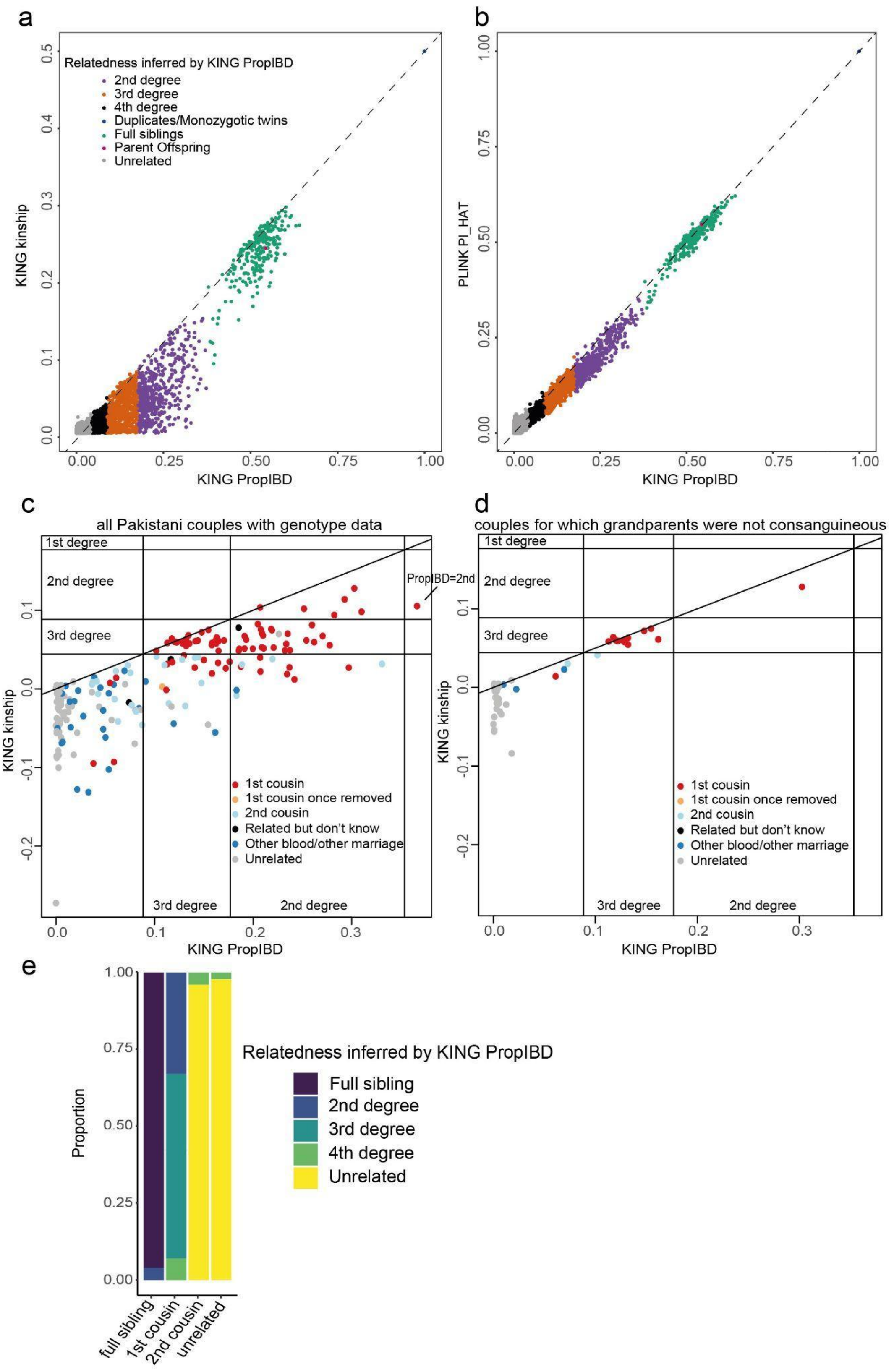

Supplementary Figure 2: Relatedness checks. a-b) Relatedness estimates between all BiB CoreExome Pakistani mothers compared using three different metrics, where the colours indicate the relationship type inferred by KING-PropIBD. a) KING-kinship versus KING-PropIBD; b) KING PropIBD versus PLINK's $\widehat{\Pi}$ (PI_HAT). In (c) and (d), we compare the two KING estimators for the 196 Pakistani couples from the GSA/CoreExome

intersection for whom we had self-declared relationship information. Plot (c) shows all couples and (d) shows only those for which both the mother's parents and father's parents were reported to be unrelated. The vertical and horizontal lines indicate the recommended cutoffs for defining relatives, and the sloped line indicates the expectation if the two methods were performing identically. (e) Breakdown of relationships inferred by KING-PropIBD between pairs of individuals simulated to have the relationship indicated on the x-axis (500 per category).

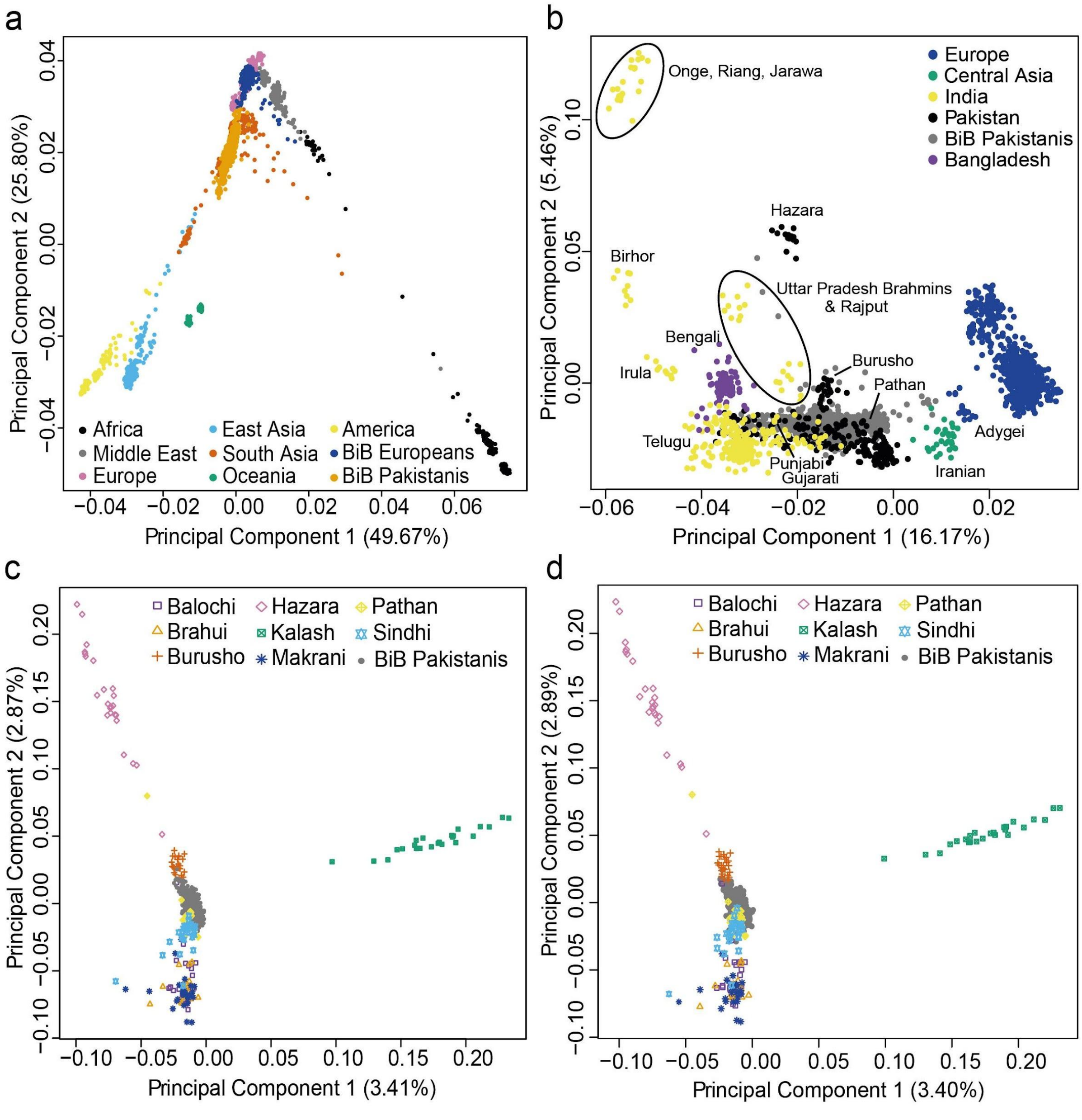

Supplementary Figure 3. PCA of Born in Bradford mothers along with external datasets. a) PCA of the HGDP dataset with BiB Pakistani and European samples projected onto it, illustrating their position in a worldwide context. b) PCA of HGDP, 1000 Genomes Project Phase 3 and published genotypes from modern samples (https://reich.hms.harvard.edu/allen-ancient-dna-resource-aadr-downloadable-genotypes-present-day-and-ancient-dna-data), with BiB Pakistani samples projected onto them. c) PCA of HGDP Pakistani samples, with BiB Pakistani samples projected onto them. d) PCA

computed using HGDP Pakistani samples and 25 BiB Pakistanis, with the remaining BiB Pakistanis projected onto them. In both (c) and (d) the BiB Pakistanis cluster together on the PCA, on top of the HGDP Pathan and between the Sindhi and Burusho

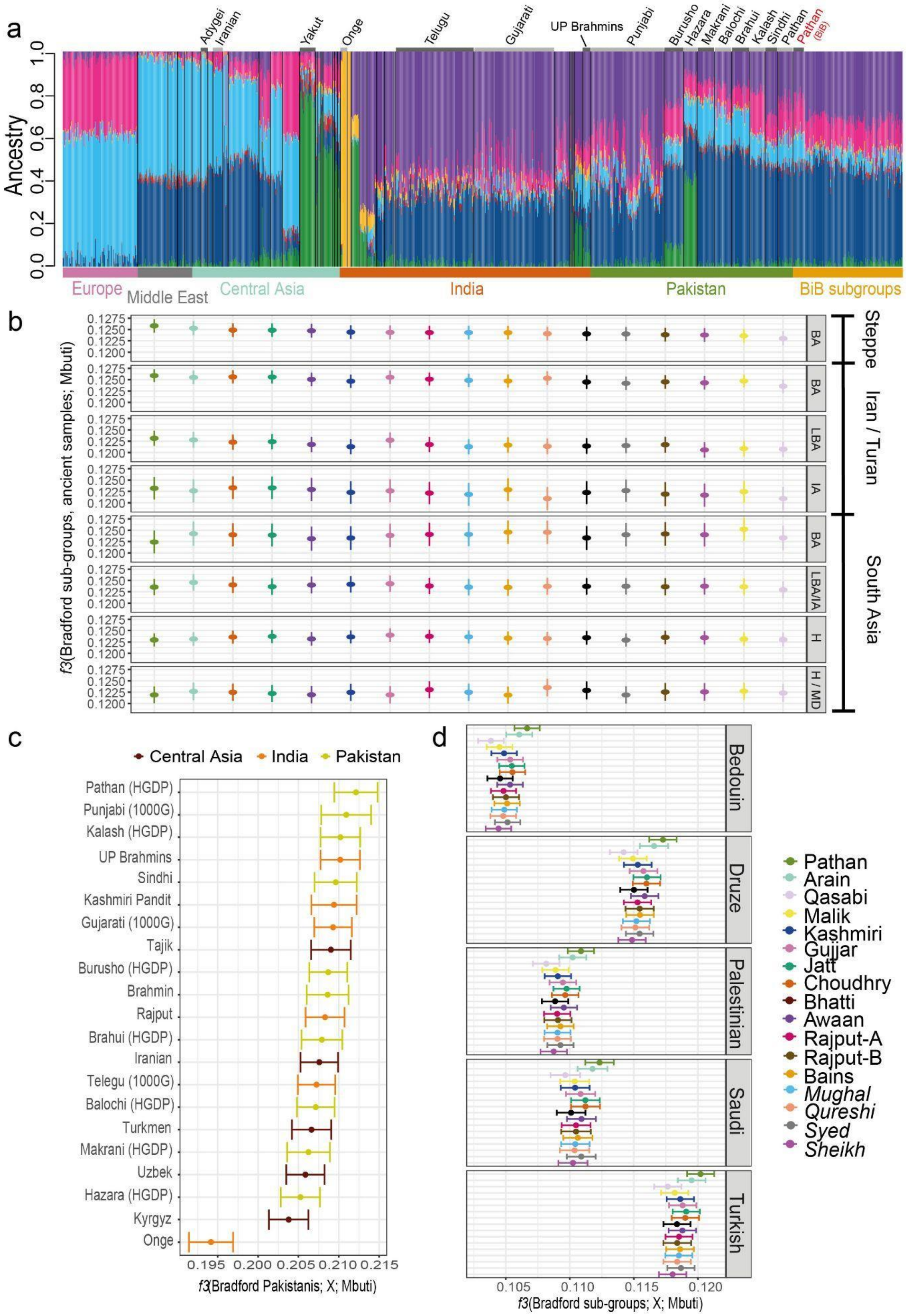

Supplementary Figure 4. Genetic similarity between Bradford Pakistanis and other worldwide populations. a) Admixture plot (K=8) of HGDP, 1000 Genomes Project Phase 3, published genotypes from modern samples (https://reich.hms.harvard.edu/allen-ancient-dna-resource-aadr-downloadable-genotypes-pre

sent-day-and-ancient-dna-data) and BiB subgroups (n=1064 individual samples). The latter have been downsampled for the plot but all 2,200 Bradford samples were included in the analysis. The plot illustrates different ancestral components making up the various worldwide populations. BiB Pathans (in red) stand out compared to other subgroups and have a similar genetic profile to the HGDP Pathans. b) Outgroup *f3*-statistics of BiB Pakistanis compared to ancient genomes from Central and South Asia. The y-axis represents the *f3*-statistics, computed with the phylogeny *f3* (Bradford sub-groups, ancient genomes; Mbuti). Each panel represents one archaeological period for the three archaeological sites considered (Steppe, Iran/Turan, South Asia). BA= Bronze Age, LBA= Late Bronze Age, IA= Iron Age, H= Historical, MD= Historical/Medieval. The error bars of the *f3*-statistics estimates are standard errors obtained using blocks of 500 SNPs c,d) Outgroup *f3*-statistics of BiB Pakistanis compared to other modern worldwide populations. The x-axis represents the *f3*-statistics, computed with the phylogeny *f3*(Bradford Pakistanis, *X*; Mbuti), where *X* represents the indicated worldwide population. The higher the value, the higher the genetic sharing between the pair of populations tested. Error bars indicate standard errors. The error bars of the *f3*-statistics estimates are standard errors obtained using blocks of 500 SNPs c) All BiB Pakistanis compared to other South and Central Asian groups. d) Self-reported subgroups compared to Middle Eastern populations indicated on the right. In *italics* are the self-reported groups that claim Arabic ancestry: Qureshi, Syed, and Sheikh.

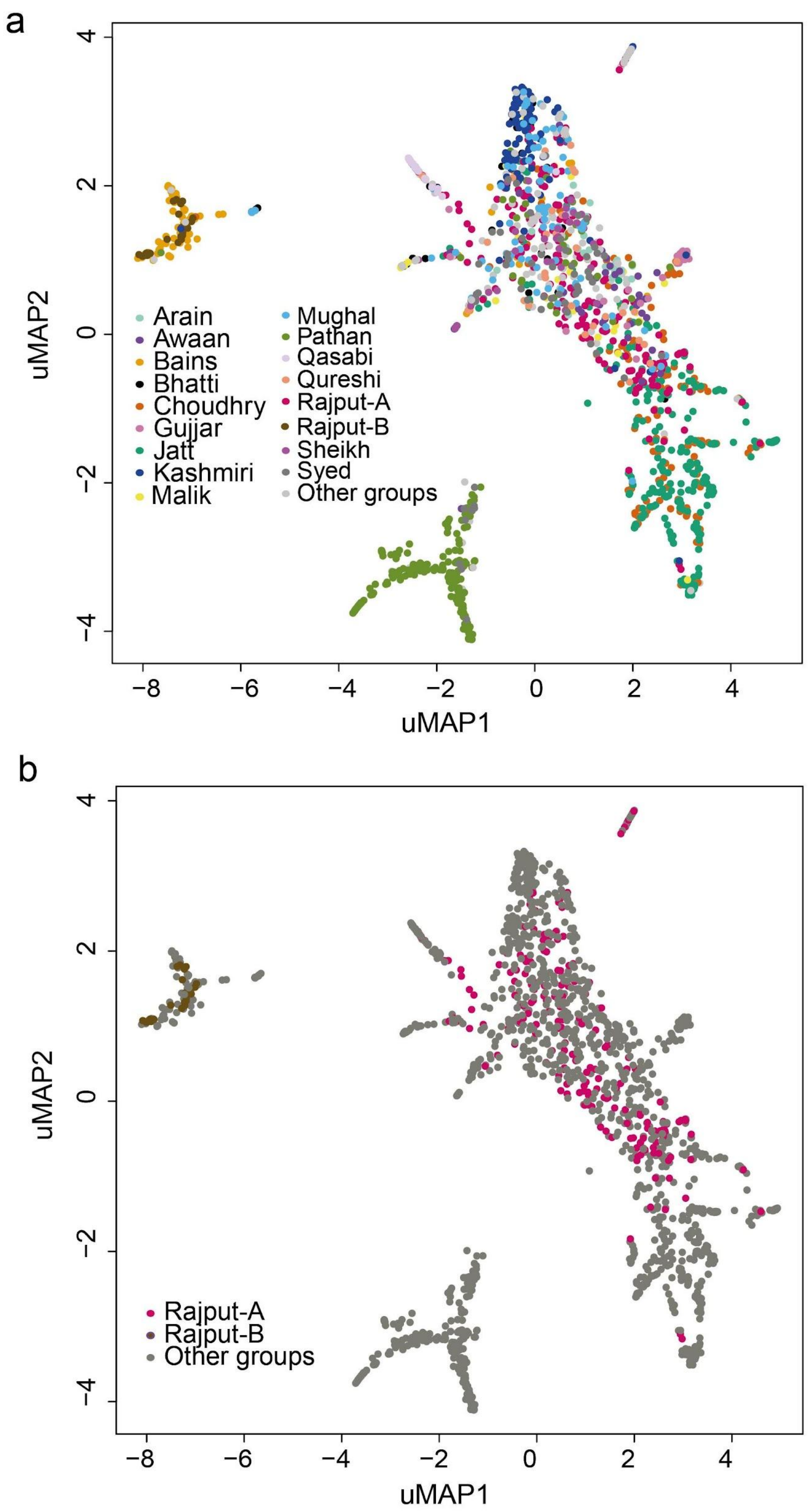

Supplementary Figure 5. Population structure of the Bradford Pakistani subgroups as inferred with UMAP analysis using 20 PCs. a) UMAP plot coloured by major self-reported groups. b) The same UMAP plot but highlighting Rajput-A and Rajput-B samples in pink and brown respectively.

a

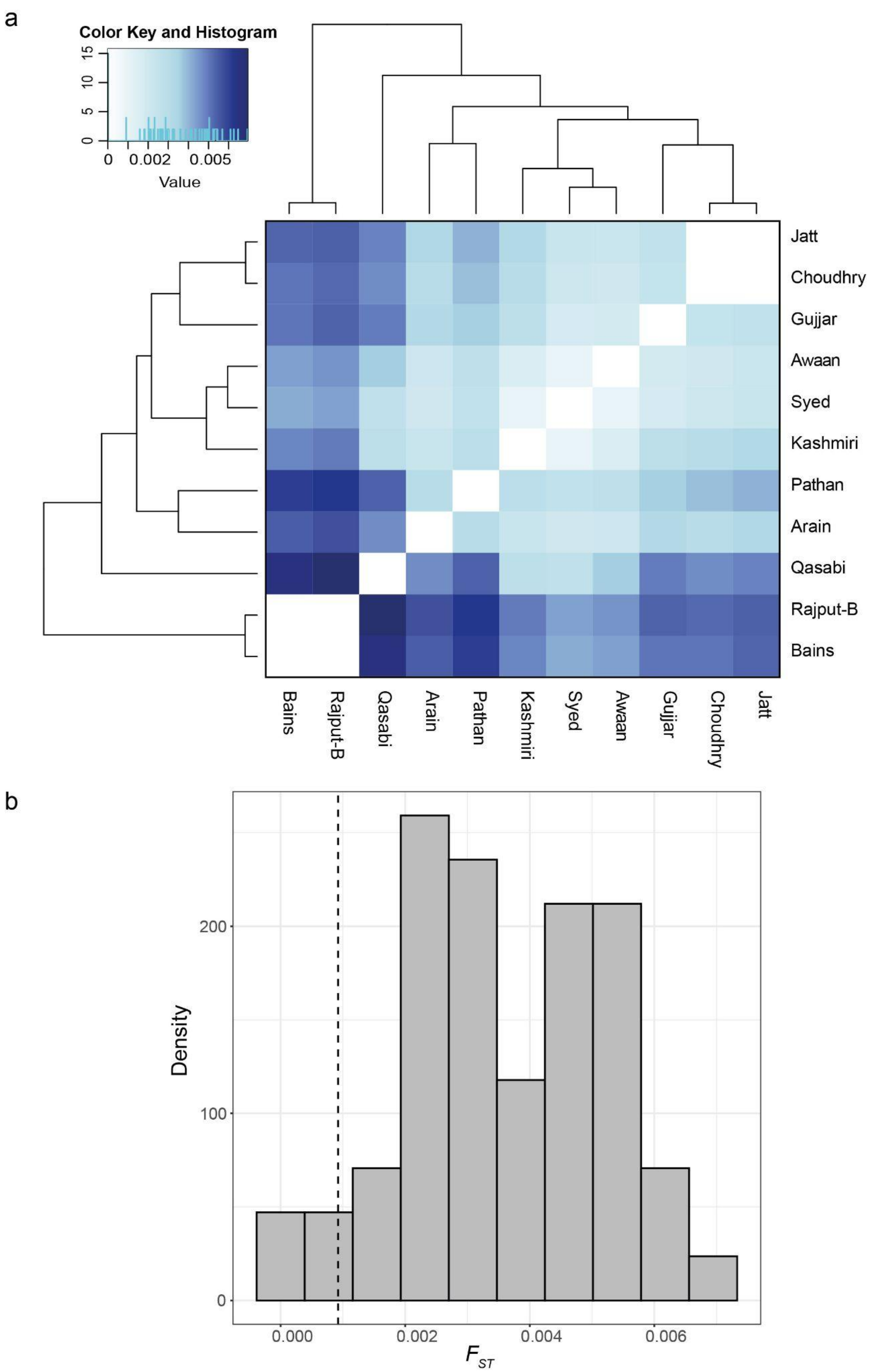

Supplementary Figure 6. a) Heatmap of pairwise F_{ST} values for the subgroups, using only individuals who fell within the dominant cluster for that subgroup on fineSTRUCTURE (see Methods). b) Distribution of all pairwise F_{ST} values, with the dashed line denoting the 5th percentile of the empirical distribution.

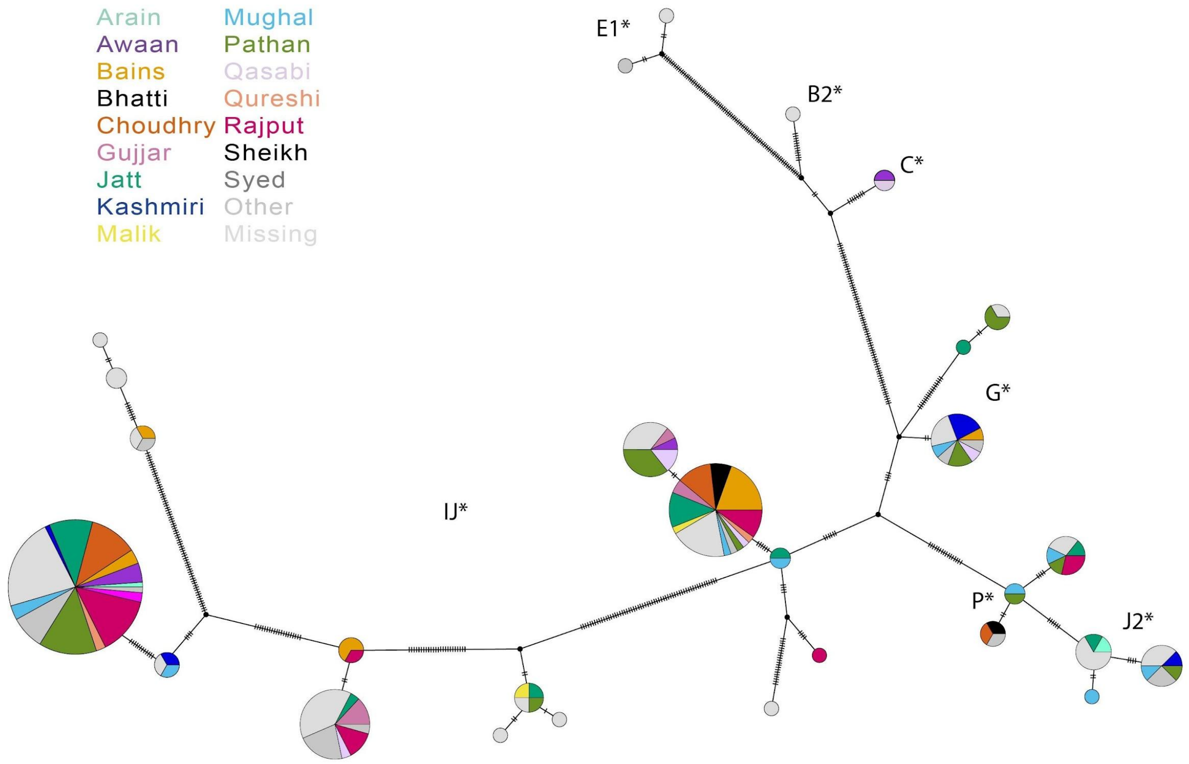

Supplementary Figure 7. Y chromosome haplotype network based on median joining for 228 BiB Pakistani fathers, constructed from Y chromosome SNPs on the GSA chip. The area of the circles is proportional to the frequency of the haplogroup. Note that the branch length is not proportional to the genetic distance. The mutations separating each haplotype are indicated as hatch marks. The letters on the plot represent the different haplogroups.

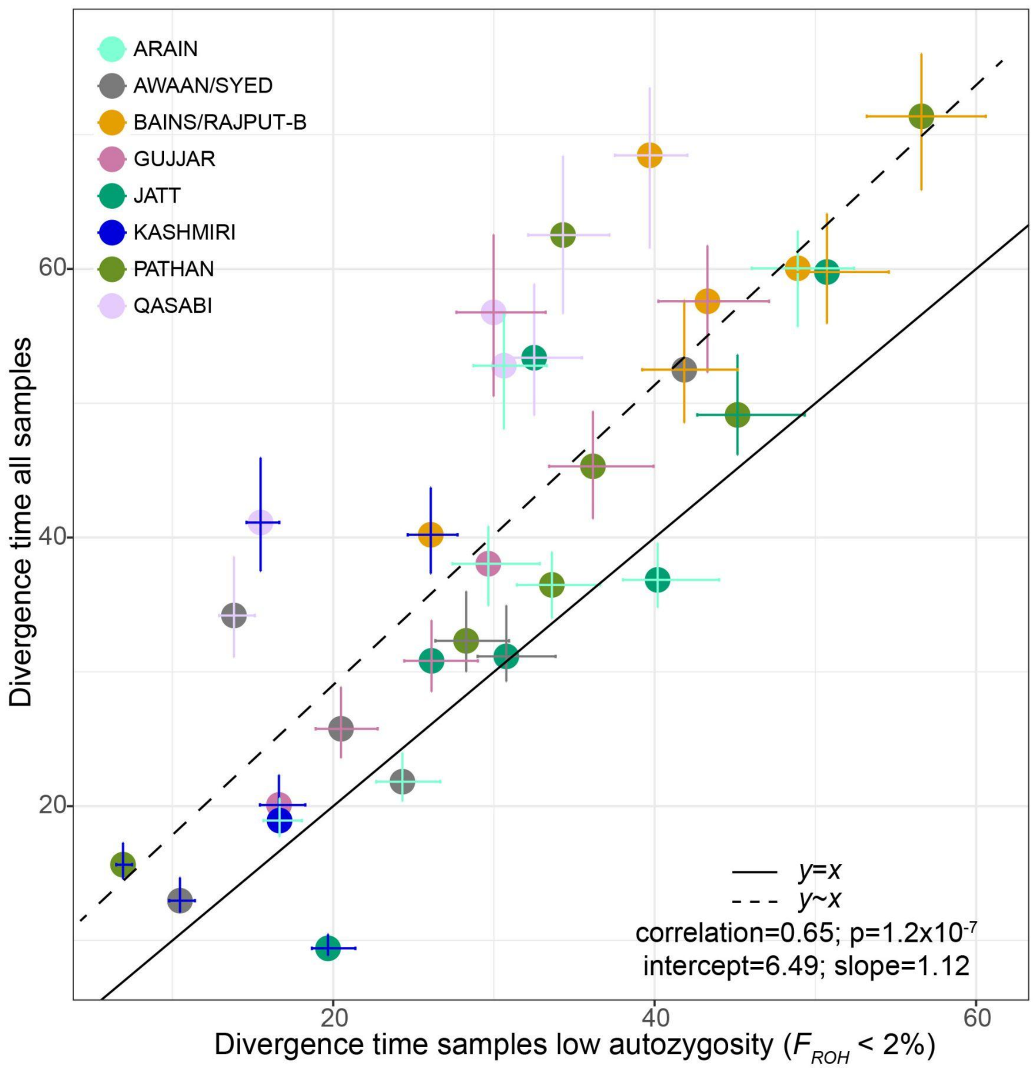

Supplementary Figure 8. Comparison of divergence times between the homogeneous subgroups using all samples versus only samples with low autozygosity (n=850 independent samples). The error bars indicate 95% confidence intervals of the divergence time estimates. The colours of the points and their 95% confidence intervals indicate the two groups whose divergence time is represented. The dashed line indicates the line of best fit generated with the linear regression (lm) function with a two-sided alternative hypothesis test. The intercept and slope are given in the legend. The adjusted p-value for multiple testing correction is reported.

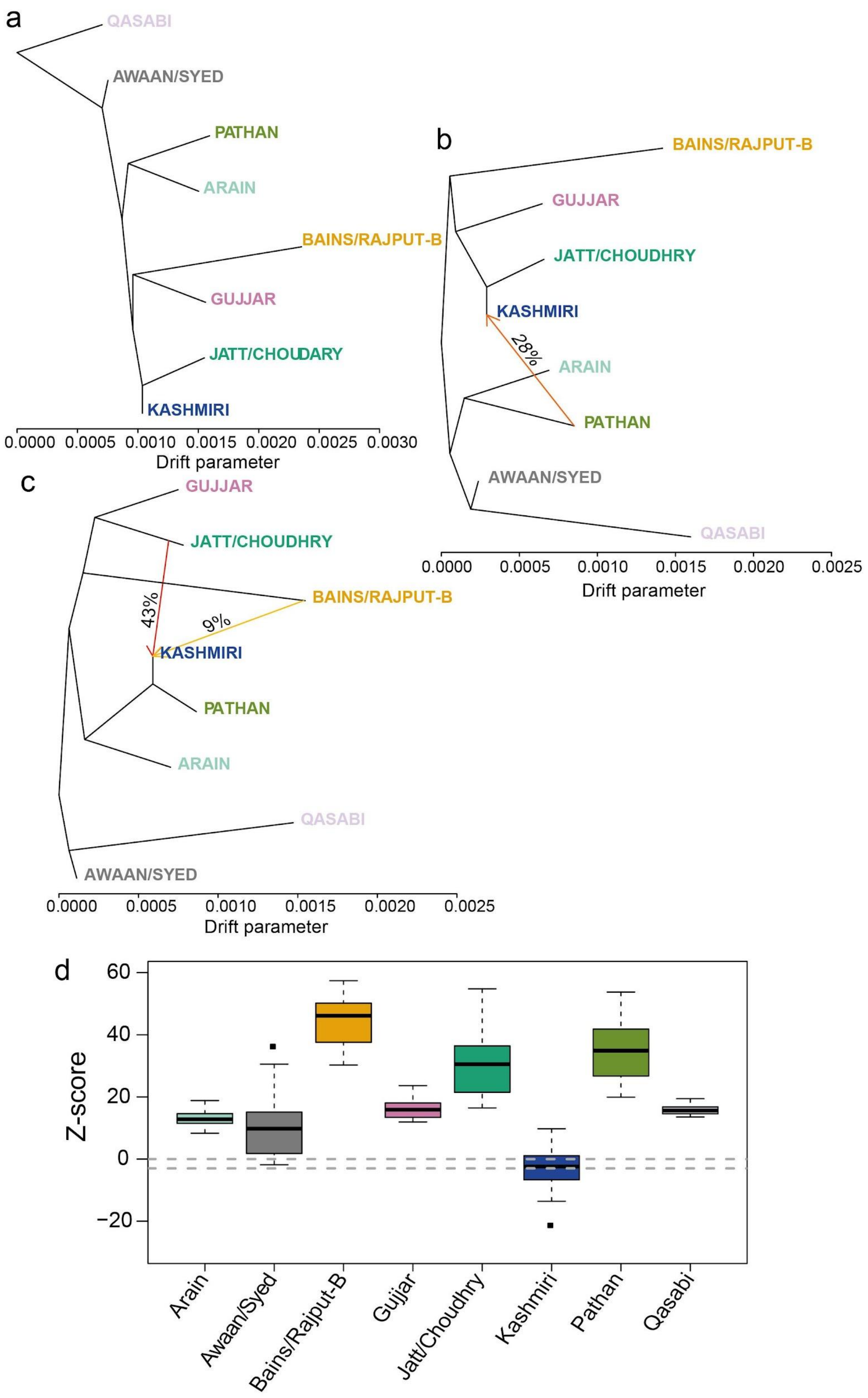

Supplementary Figure 9. Inferred population trees with different mixture events based on Treemix analyses, using the homogeneous Pakistani subgroups defined with fineSTRUCTURE. The migration arrows are coloured and labelled according to their weight,

which is correlated with the ancestry fraction shared. a) No migration edges allowed. b) One migration edge allowed. c) Two migration edges allowed. This could indicate, for example, that, according to the tree topology shown in (c), 43% of Kashmiri ancestry is derived from Jatt/Choudhry and 9% from Bains/RajputB. Note that the tree topology changes as migration edges are added, so one should not place too great a weight on it. d) Boxplot of *f3*-statistics test of admixture, using all possible combinations of sources among the homogeneous Pakistani subgroups defined with fineSTRUCTURE (n=850 independent samples). Dashed lines represent Z-score values of 0 and -3 which represent indication of putative admixture and significant admixture respectively. The only population that shows significant *f3* values is the Kashmiri. In each boxplot the centre is equal to median, the upper and lower bounds of the box correspond to 25th and 75th percentiles, and the whiskers represent 1.5 times the inter-quartile range (IQR) from the bounds of box. The outliers are represented as points.

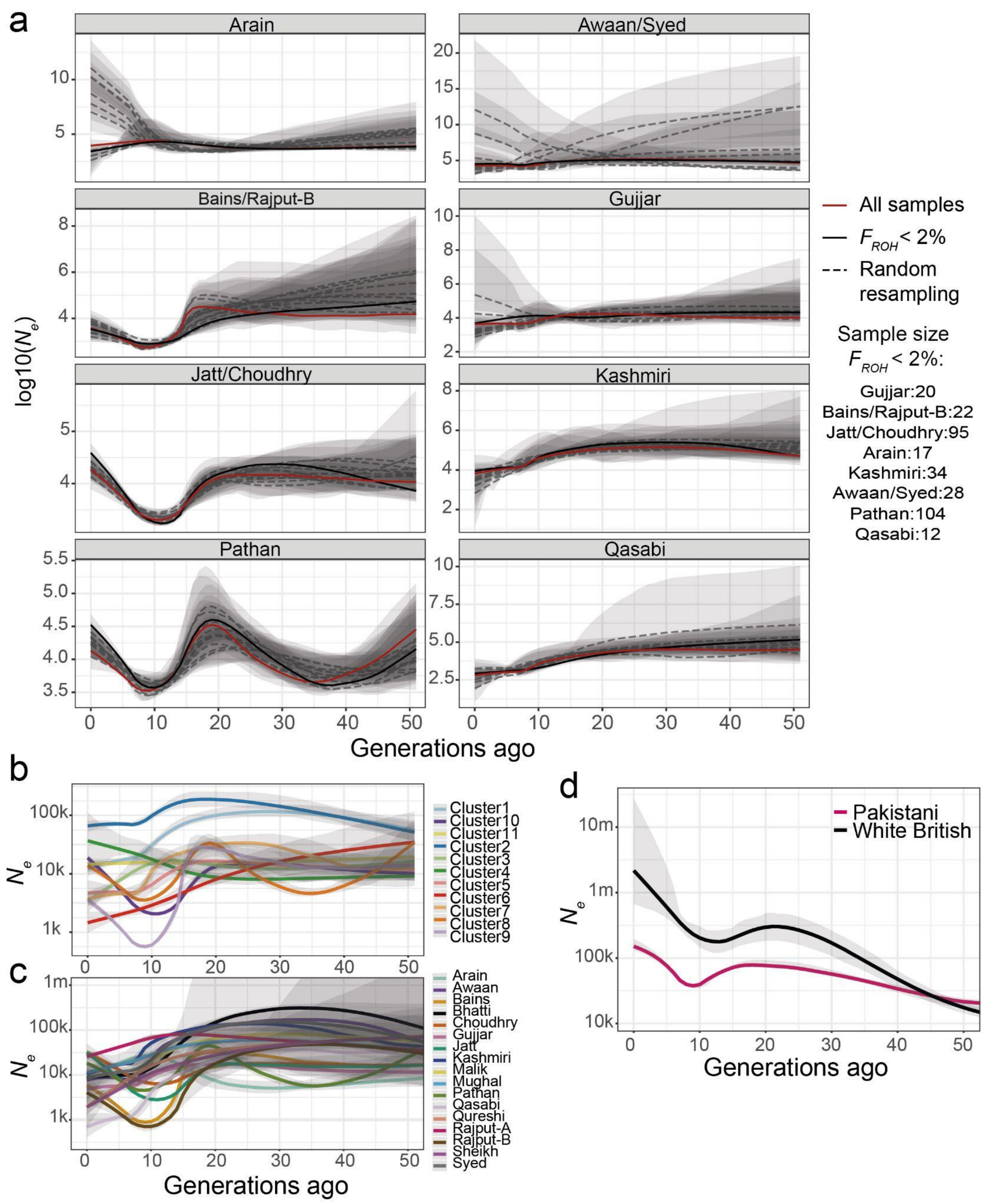

Supplementary Figure 10. Effective population size (N_e) changes through time estimated with IBDNe using different subsets of samples. The solid and dashed lines indicate mean estimates, and the grey shading indicates 95% confidence intervals for the N_e estimates. a) Comparison of all samples (red solid line), samples with low autozygosity (F_{ROH}<2%) (solid black line), and random downsampling from the set of all samples (grey dashed lines) for each homogeneous Pakistani subgroup defined by fineSTRUCTURE. Samples were randomly downsampled 10 times from all samples to match the sample size of the low autozygosity set indicated in the legend. b) N_e estimates for all fineSTRUCTURE clusters (shown in Figure 2). c) N_e estimates for the major self-reported groups. d) Comparison of mean estimates when analysing all BiB Pakistanis and White British.

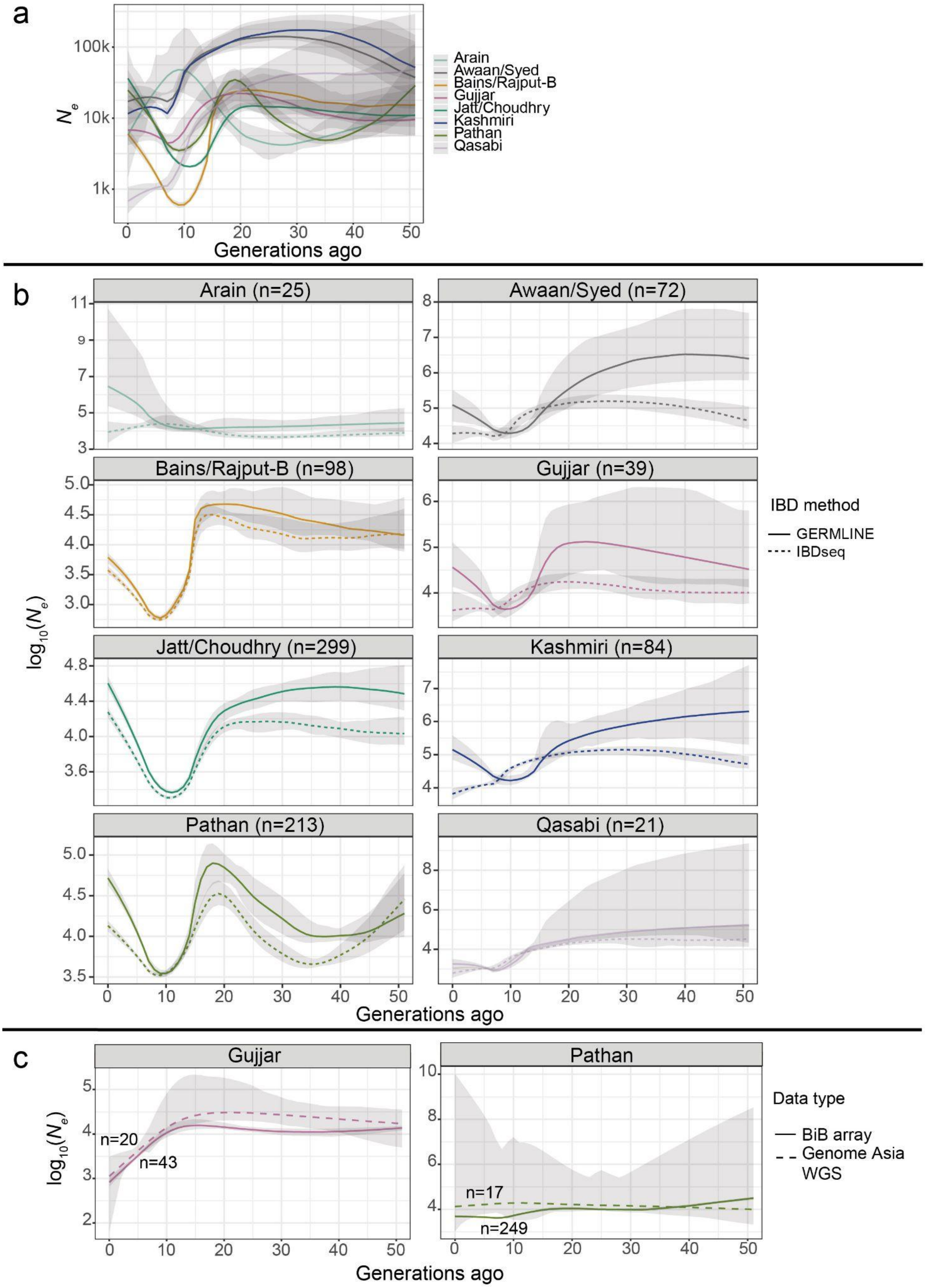

Supplementary Figure 11. Change in effective population size (N_e) through time estimated with IBDNe using different IBD calling methods, data types or filtering methods. The solid and dashed lines indicate the mean estimates, and the grey shading indicates 95% confidence intervals of the N_e estimates. a) Mean estimates for Pakistani homogeneous subgroups using unfiltered IBDSeq IBD calls. b) Comparison between the mean estimate

using GERMLINE IBD output (solid line) and using IBDSeq output (dashed line). The sample size for each subgroup is indicated in brackets. c) Comparison between the mean estimate using BiB Core exome array samples and Genome Asia v1 whole-genome sequencing samples with the sample sizes indicated on the plot.

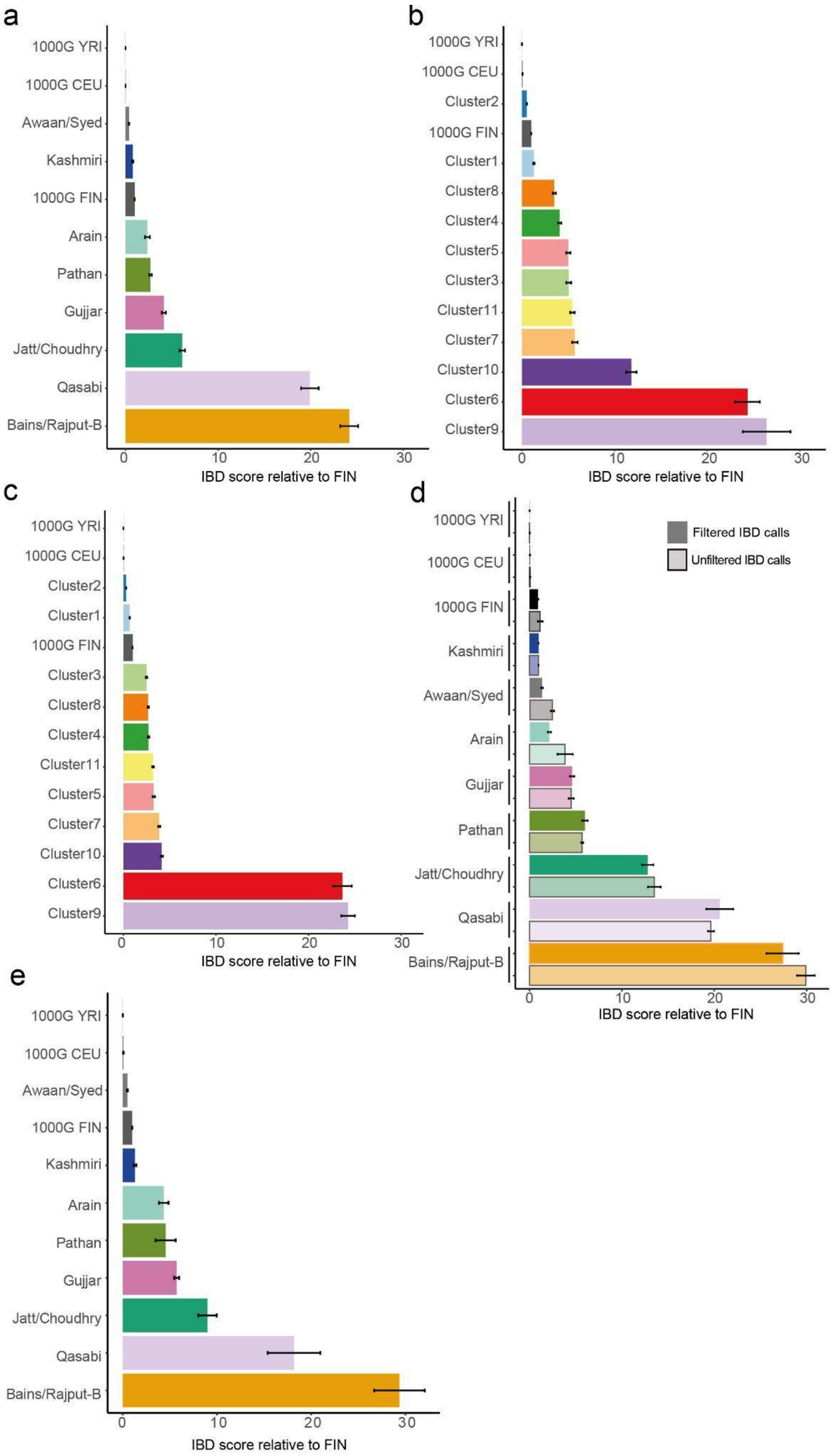

Supplementary Figure 12. IBD scores calculated for individuals from the indicated group standardised by the value for the 1000 Genomes Finnish individuals. The error bars indicate the IBD score standard error. a) Homogeneous groups defined by fineSTRUCTURE considering the total length of IBD segments between 3 and 20cM, i.e. the same cutoffs

used in Nakatsuka *et al.* [20] (n=912 independent samples) b-c) All genetic clusters defined by fineSTRUCTURE, considering the total length of IBD segments between 5 and 30cM (i.e. the filtering used for Figure (n=1583 independent samples) 4a) (b) or between 3 and 20cM (c) (n=1401 independent samples). d) Homogeneous groups defined by fineSTRUCTURE using filtered and unfiltered IBD calls (see Supplementary Note 1). Filtered calls have been used for the panels a-c and Figure 4a (n=964 independent samples). e) Homogeneous groups defined by fineSTRUCTURE considering the total length of IBD segments between 5 and 30cM using IBDseq IBD calls (n=923 independent samples).

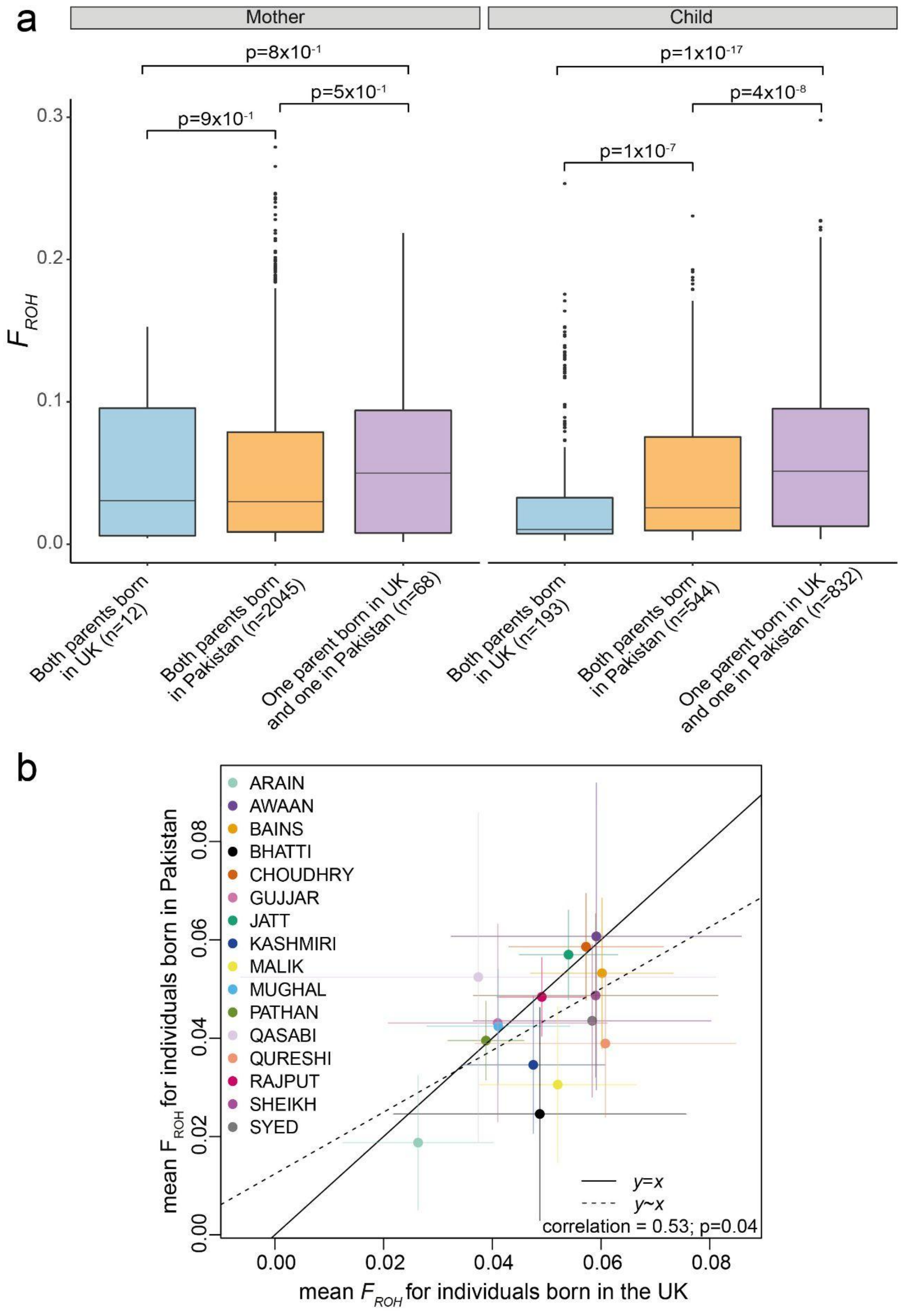

Supplementary Figure 13. Patterns of F_{ROH} in BiB children and mothers according to parents' or own birthplace. a) Boxplot of F_{ROH} for Pakistani mothers and children split by their parents' birthplace. In each boxplot the centre is equal to median, the upper and lower bounds of the box corresponds to 25th and 75th percentiles, and the whiskers represent 1.5

times the inter-quartile range (IQR) from the bounds of box. The outliers are represented as points. The outliers are represented as points. P-values are from two-sided Wilcoxon tests. b) Average F_{ROH} with 95% confidence intervals for Pakistani mothers born in Pakistan versus in the UK, split by self-declared subgroup. The slope of the line of best fit (dashed line) is 0.63 (standard error 0.27) (p=0.04). Only the Malik show a significant difference between those born in Pakistan versus the UK (p-value from one-sided t-test = 0.03).

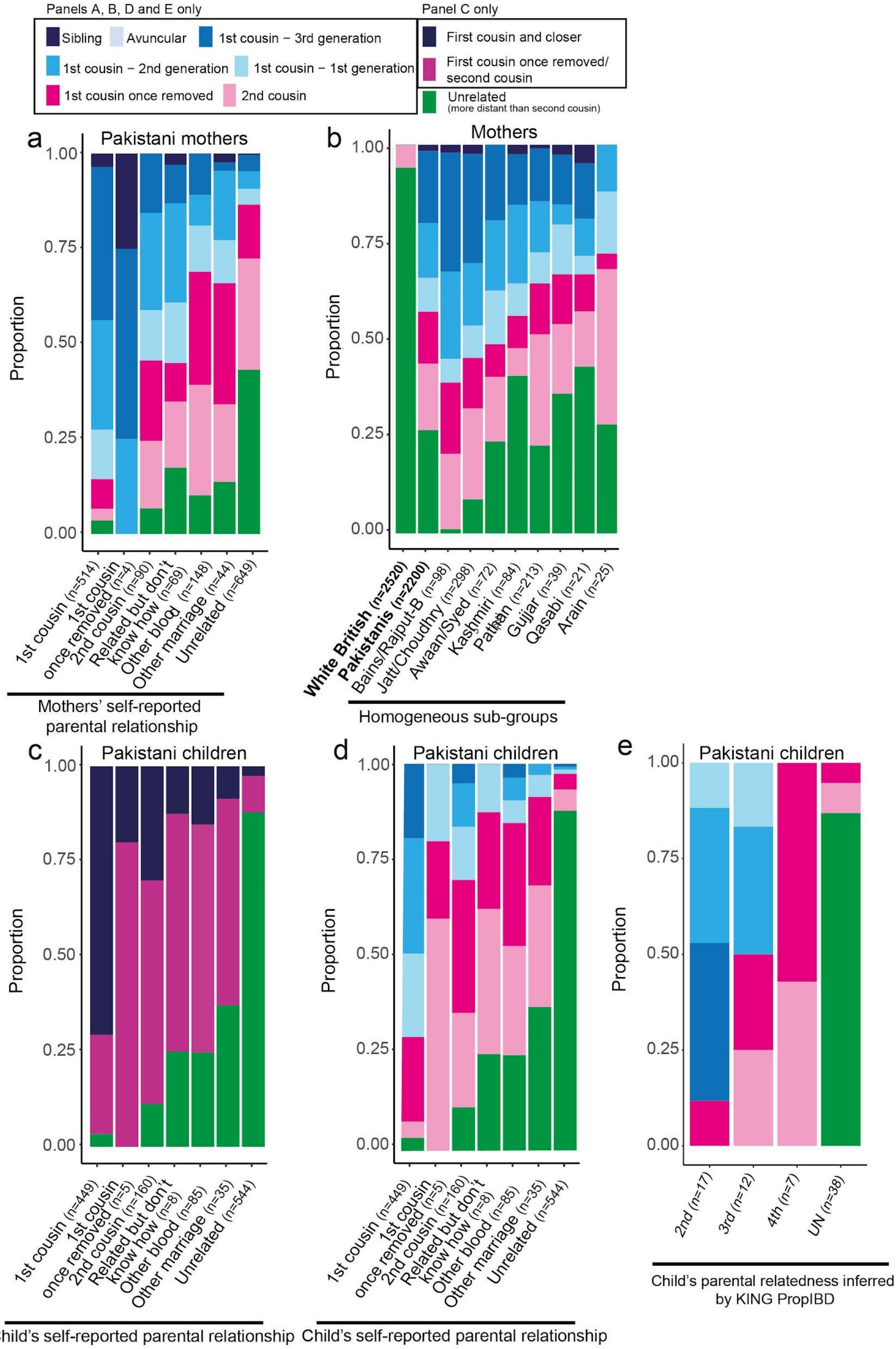

Supplementary Figure 14. Inferred parental relationships for BiB mothers and children. a-b) Stacked bar plots showing inferred parental relatedness for the mothers broken down by (a)

reported parental relatedness or (b) subgroup. Note that the various avuncular relationships have been combined into one category since they accounted for very few individuals. c-d) Stacked bar plots showing inferred parental relatedness for the children broken down by reported relationship between the mother and her husband, showing either the three broad categories (c) or all ten categories (d). e) Stacked bar plot showing the inferred parental relatedness for the children broken down by KING PropIBD inferred relatedness between the mother and her partner. Note that the model included three classes of avuncular unions (over one, two or three generations) but these have been combined into one in these figures since very few individuals were inferred to be offspring of avuncular unions.

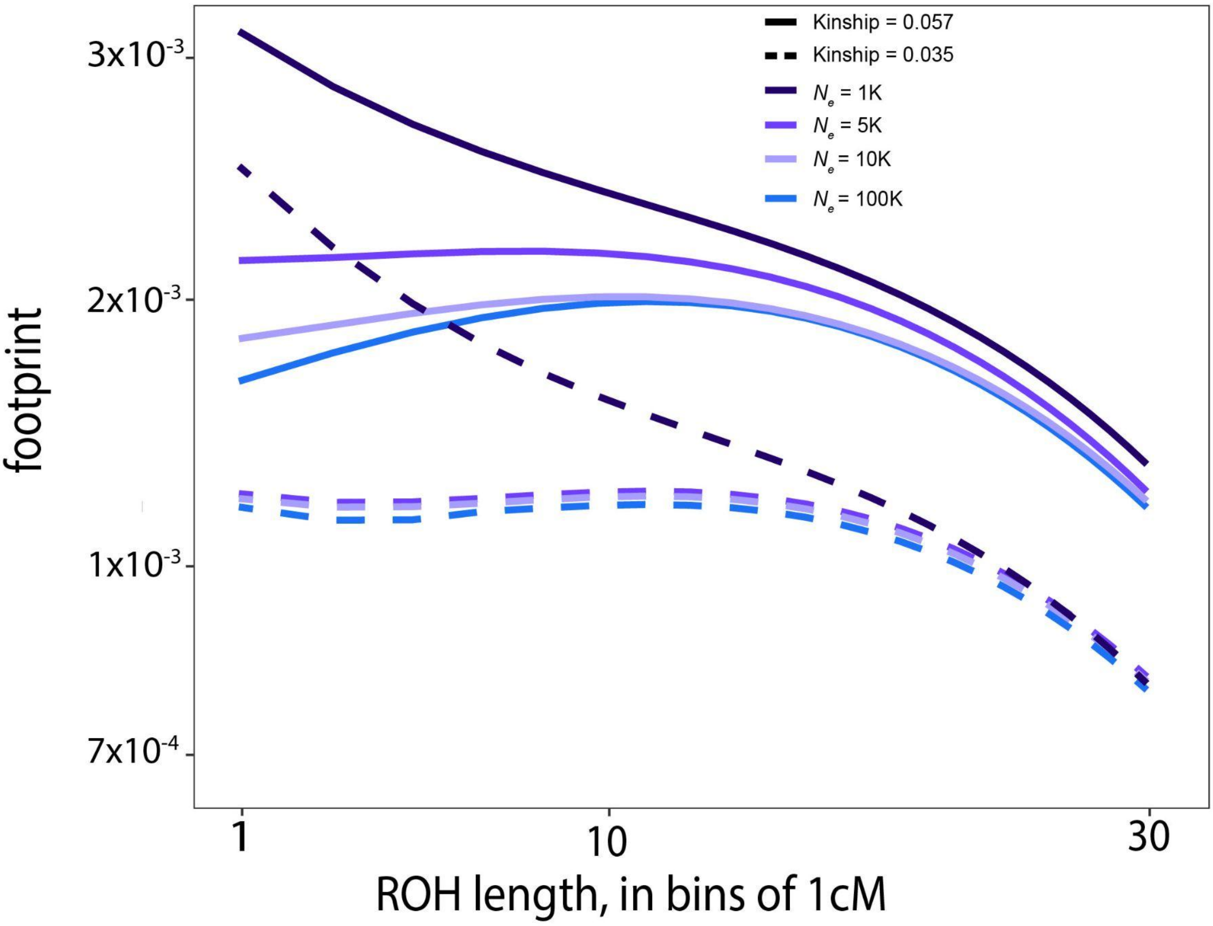

Supplementary Figure 15. Impact of the effective population size and average kinship between spouses on the expected ROH footprint determined using the theory from [27] and Methods. The ROH footprint is the average fraction of the genome covered in ROHs of a given length. N = number of reproducing couples (i.e. $N_e/2$). The average kinship values shown represent our inferred estimates for Pathan (0.035) and Jatt/Choudhry (0.057). Very low N_e has a major influence on the expected footprint of ROHs < 10cM.

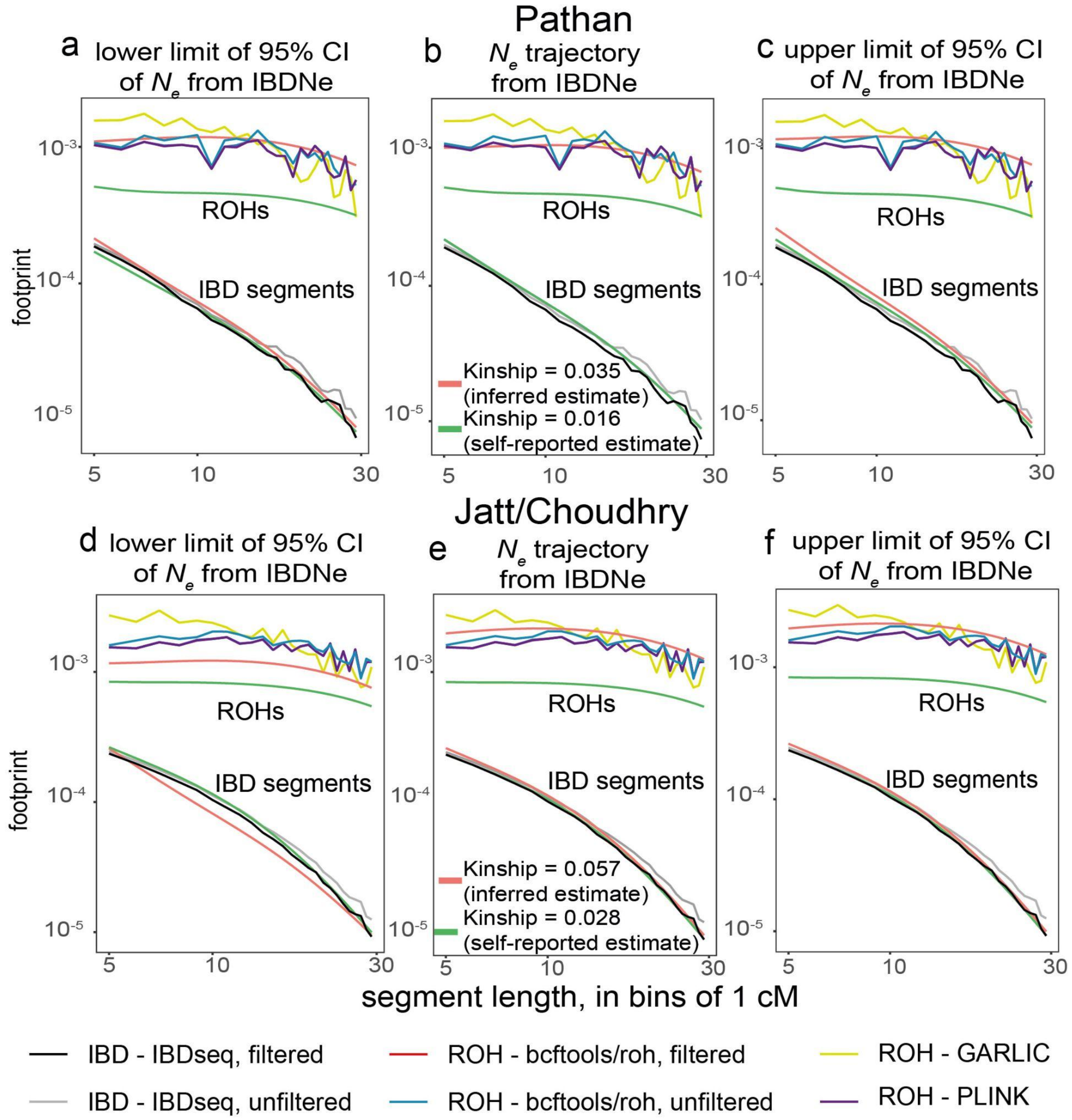

Supplementary Figure 16. Observed ROH and IBD footprints compared to expectation under the model in [27]**.** The footprint is the average fraction of the genome covered by segments of a given length interval. The upper lines represent the ROH footprint and the lower lines the IBD footprint. Points are plotted at the beginning of each 1cM interval. The expectation was determined using the indicated kinship values and either the point estimates of $N_e(t)$ from IBDNe (middle plots) or the lower or upper bound of their 95% confidence interval for $t \leq 50$ (left hand and right hand plots, respectively), then a constant N_e for $t > 50$. The IBDNe results used were for Pathan from fineSTRUCTURE Cluster 8 (a-c) or Jatt/Choudhry from Cluster 10 (d-f), and the corresponding observed footprints are plotted accordingly. We show the observed IBD footprint from IBDseq calls and the observed ROH footprint with bcftools/roh calls both with and without the filtering described in Methods, as well as the ROH footprint from PLINK and GARLIC ROH calls.

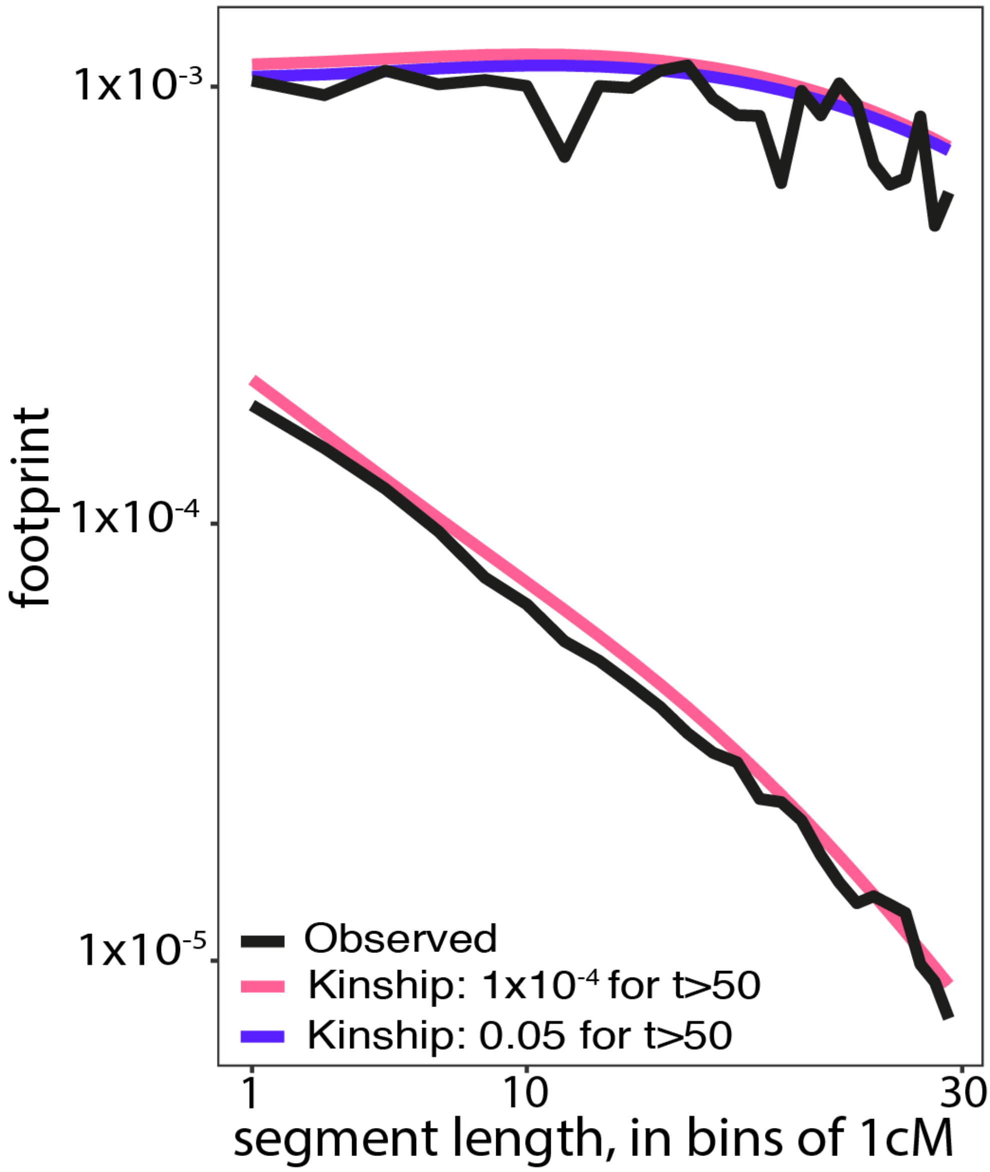

Supplementary Figure 17. Effect of changes in historical consanguinity rates on the expected ROH and IBD footprints. The footprint is the average fraction of the genome covered by segments of a given length interval. The upper lines represent the ROH footprint and the lower lines the IBD footprint. Points are plotted at the beginning of each 1cM interval. The expectation was determined using the mean IBDNe estimate as $N_e(t)$ for Pathan from fineSTRUCTURE cluster 8 and an average parental kinship value, *k*, of 0.035 for $t \leq 50$, then using a constant N_e and the indicated *k* for $t > 50$. Note that the expected ROH footprint is barely altered when using *k*=1x10^{-4} versus *k*=0.05. The observed IBD footprint comes from IBDseq calls and the observed ROH footprint from bcftools/roh calls, with the filtering described in Methods.

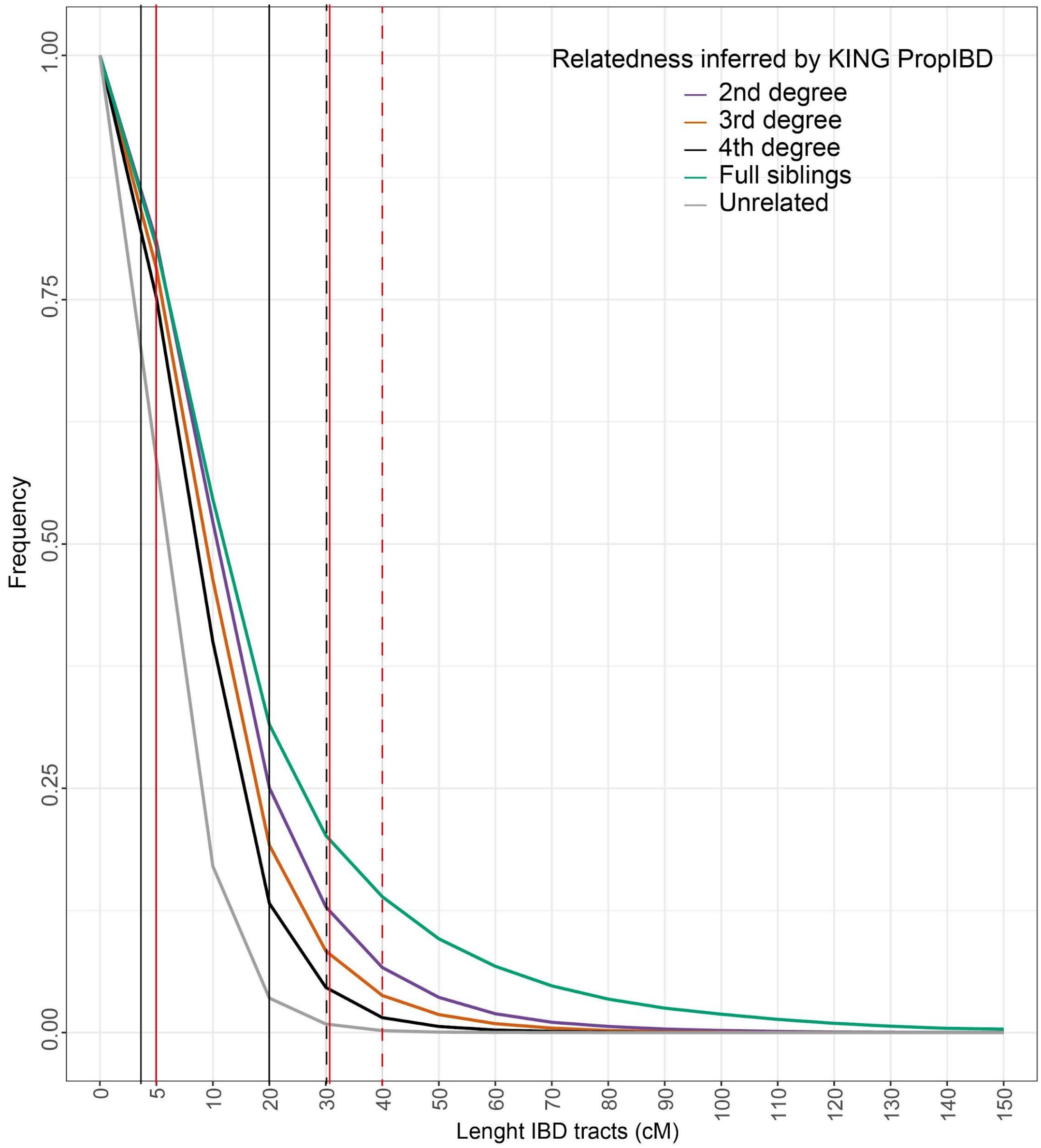

Supplementary Figure 18. Cumulative distribution of IBD segments called by GERMLINE stratified by KING coefficient of relationship (PropIBD). The plot shows the proportion of individuals who share at least one segment greater than the length bin indicated on the x-axis, stratified by coefficient of relationship. The vertical lines represent the IBD thresholds used for IBD scores (see Methods, Figure 4): the dashed lines represent the thresholds used to define and exclude possible relatives and the full lines represent the range of IBD considered for the IBD score calculations. Red lines indicate those used for Figure 4, and black lines those used in Nakatsuka *et al.* and for Supplementary Figure 15a.

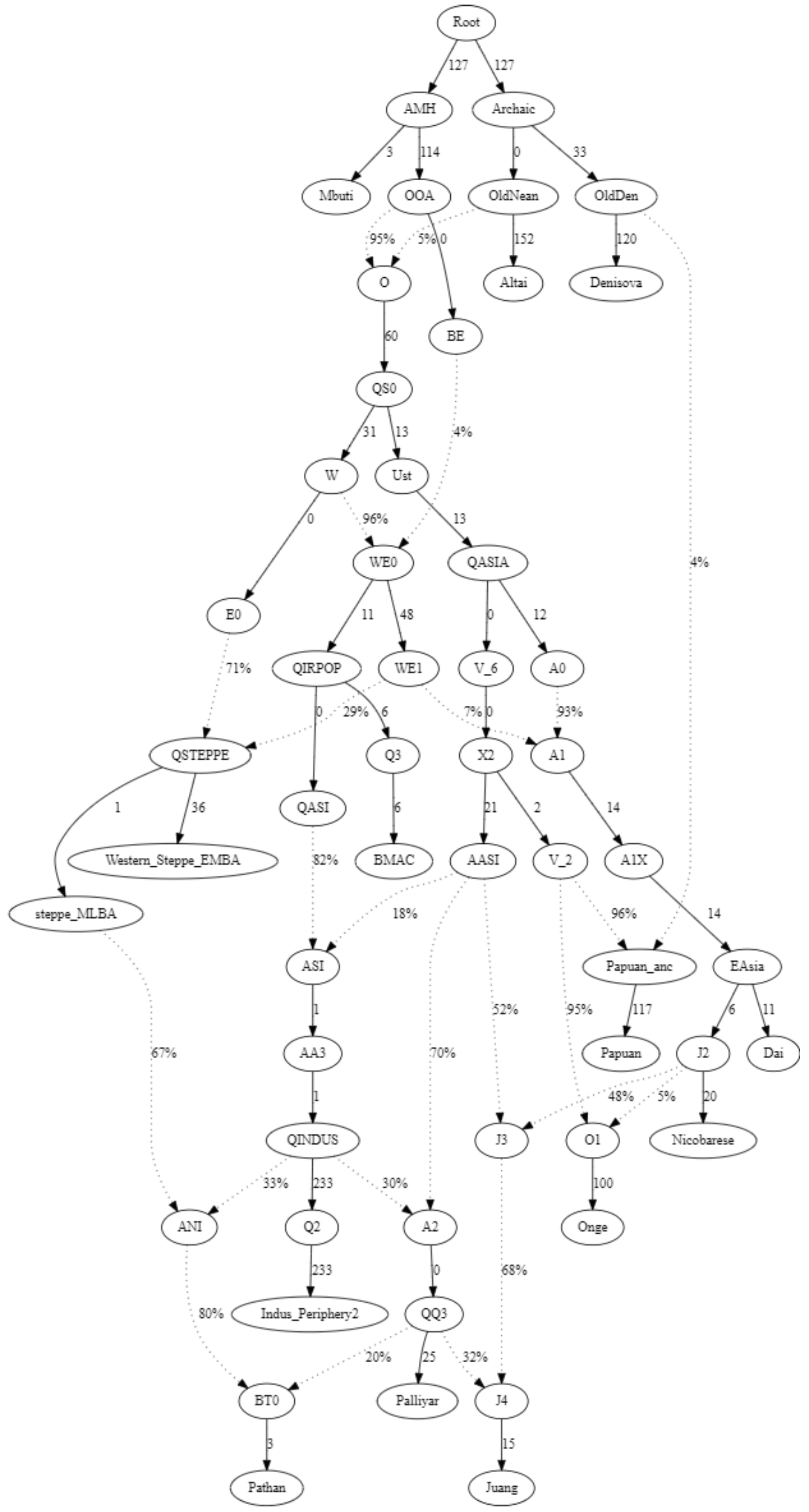

Supplementary Figure 19. Qpgraph admixture graph model. The graph shows the modelling of Bradford subgroups (represented by Pathan) as a mixture of ANI and ASI ancestral components as seen in [3]. Admixture events are represented by dotted lines labelled with the percentage of admixture. In this graph, Pathan is characterised by 80% of ANI and 20% of ASI ancestries. The largest deviation between theoretical and empirical *f*-statistics was |Z-score|= 2.9, suggesting a good fit of the model considering the vast

number of *f*-statistics analysed. Results for all other Bradford subgroups are in Supplementary Data 15.

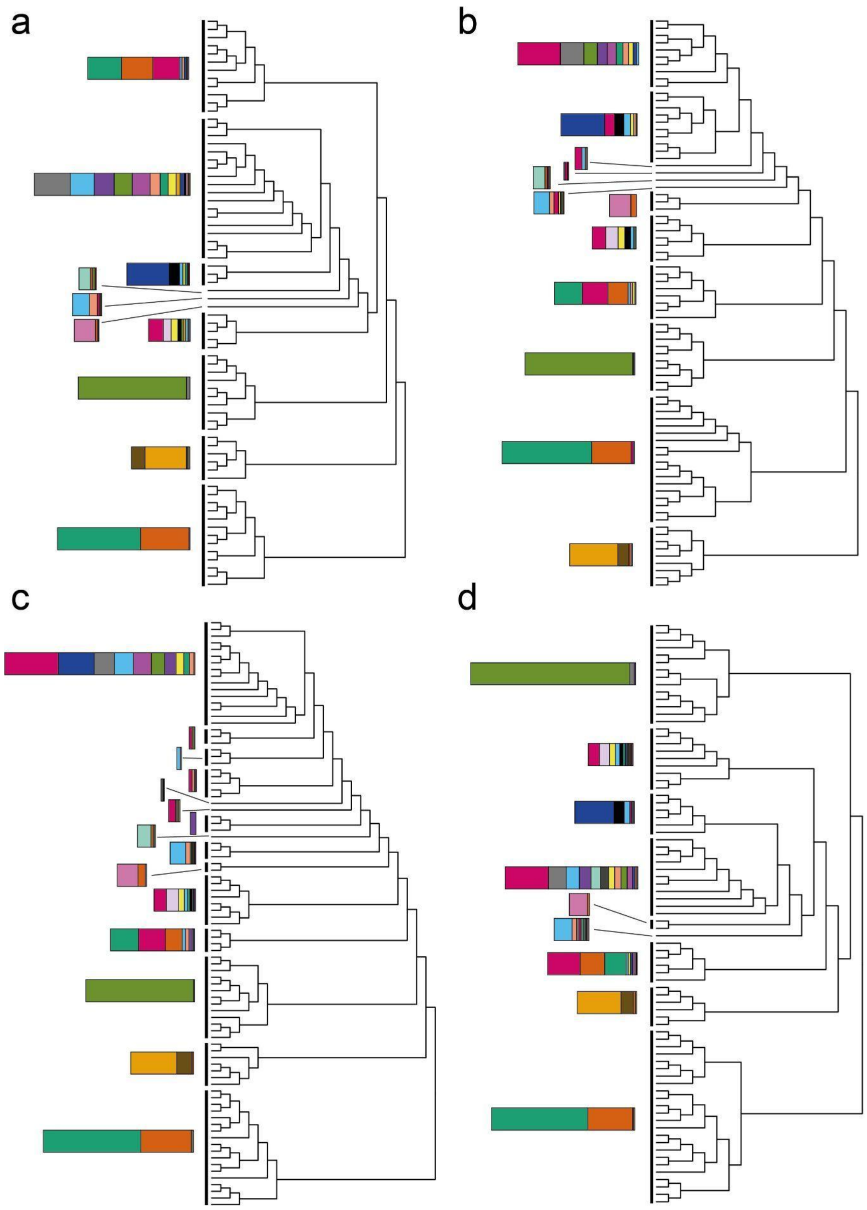

Supplementary Figure 20. Robustness of fineSTRUCTURE results from the downsampling approach. The trees (a-d) illustrate the results of hierarchical clustering of the co-ancestry matrix using patterns of haplotype sharing from ChromoPainter using a random sample (60%) of each cluster shown in Figure 2a . Each ‘leaf’ on the tree contains multiple individuals. The coloured horizontal bars represent the composition of each of the major clusters contained within the adjacent clades (delineated by the black vertical lines). The length of each coloured bar is proportional to the number of individuals in that cluster, with the proportion of each colour representing the fraction of individuals from each self-reported subgroup that make up the cluster. See Figure 2 for the legend.

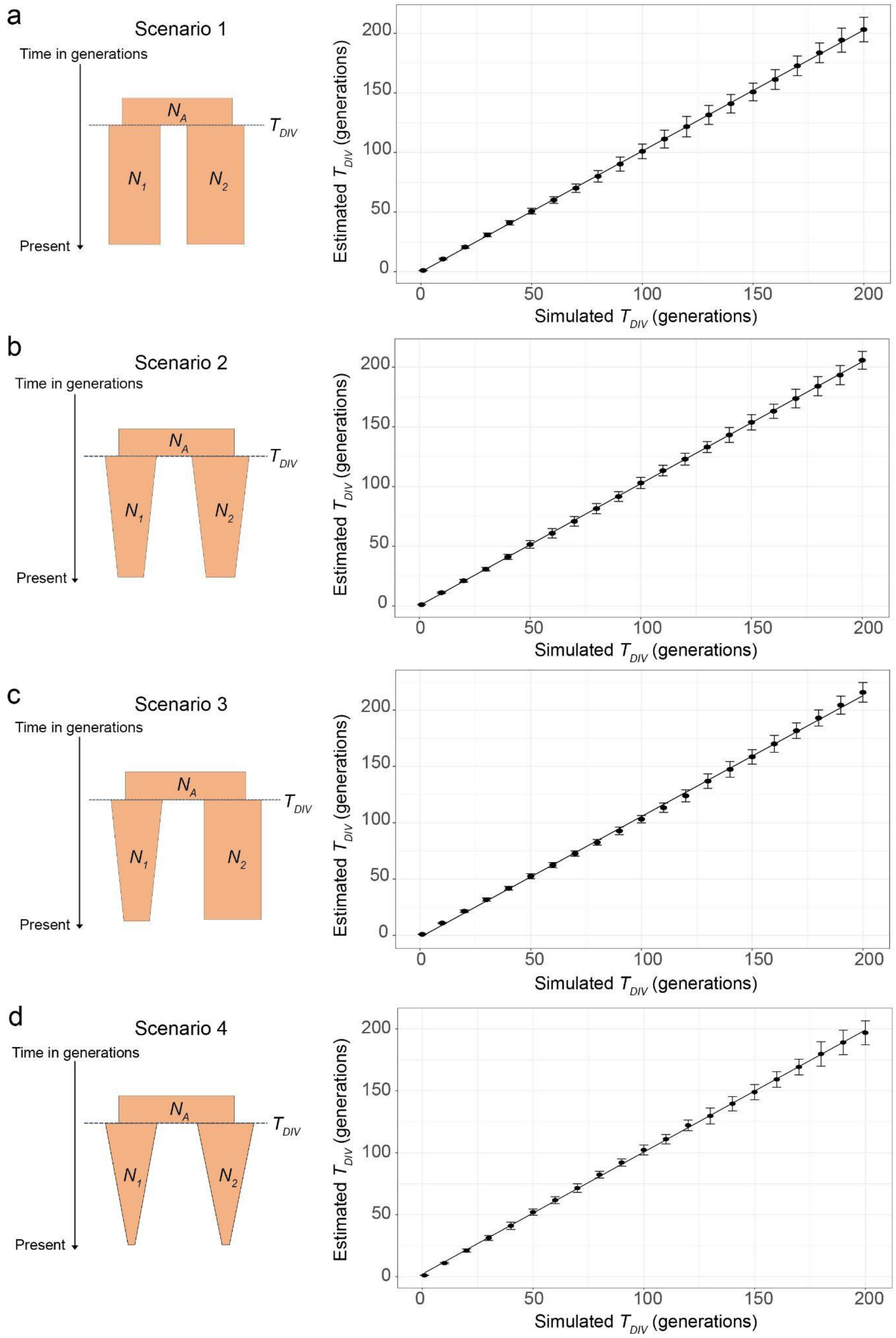

Supplementary Figure 21. Time of divergence inferred with NeonR in data generated by forward simulations. The plots show the comparison between simulated and estimated

divergence times (T_{DIV}) in the last 200 generations under different demographic scenarios (Scenarios 1-4; see Supplementary Note 1). The black dots represent the mean estimates across 10 replicates and the error bars are the standard deviations from the mean. We assumed N_A=11,000, N_1=5,000 and N_2=6,000, respectively. a) N_1 and N_2 show constant size through time. b) N_1 and N_2 show reduction in size through time (60% reduction). c) N_1 shows a reduction and N_2 shows a constant size through time. d) N_1 and N_2 show reduction in size through time (90% reduction).

Supplementary Note 2

Some key questions and answers to accompany the paper "Fine-scale population structure and demographic history of British Pakistanis" by Arciero *et al.*

1. Who conducted this study?

The study was conducted by a team of international researchers in the fields of genetics, epidemiology and anthropology. Most of the team are based at the Wellcome Sanger Institute near Cambridge or in Bradford/Leeds, both in the United Kingdom.

2. Why did you conduct this study?

Most genetic research so far has been conducted on European-ancestry individuals, leading to unprecedented knowledge of human genetic variation and population structure and history. However, many findings about the genetic contribution to disease in Europeans may not be the same for people of other ethnicities. This is because populations from different geographical areas had different historical patterns of growth and migration, and different marriage patterns, and these have affected their genetic make-up. Research into a population's genetic demographic history, structure and dynamics can give information that can be compared with information about the populations' social history, structure and dynamics. Genetics can give us information about this which can be compared with historical records. Also, a population's unique social structure and cultural history – such as the biraderi system, and the practice of consanguineous marriage – can influence its genetic population structure and genetic variability (See the Glossary at the end of this paper for more detail on terms used). Such population effects must be taken into account when searching for genes that influence disease risk, whether these be rare genetic variants that lead to severe birth defects (as considered here) or common genetic variants carried by a large proportion of the population that can increase the risk of common diseases such as heart disease or diabetes. By shedding light on the genetic make-up of the British Pakistani population, our study aims to contribute towards the drive for more personalised medicine to target the needs of specific individuals and also population-specific medicine that takes into account similarities

between members of the same group. Both these approaches have the potential to reduce health inequalities.

Questions 3 - 6 cover a basic introduction to concepts in genetics which are necessary to understand our study. The study itself is described further in questions 7 - 11.

3. What is DNA?

DNA or deoxyribonucleic acid is a complex molecule that carries our unique genetic information. During reproduction, the genetic information encoded in the DNA is passed from adult organisms to their offspring. In each cell in our body, DNA is packed up into structures called chromosomes. Humans have 46 chromosomes, made up of 23 pairs, in their cells. One set of 23 chromosomes is inherited from our mother and another set of 23 chromosomes from our father. DNA is made up of two long chains that spiral around each other to form a pattern that is described as a double-helix. These chains contain our genetic instructions coded by four chemical ‘bases’: adenine (A), thymine (T), cytosine (C), and guanine (G). Human DNA carries around 3 billion bases. The order of these bases, also called the genetic sequence, dictates the instructions for building our bodies and making them function. Information influencing specific characteristics, such as eye colour or cholesterol levels, is carried by genes that are short segments within the DNA molecule.

4. What are genetic variants?

The genome of all humans is about 99.9% identical. The remaining 0.1% holds the differences that make us unique. The term “genetic variant” refers to a specific region of the genome which can differ between the genomes of two individuals. This could be a single base in the DNA (e.g. at a particular position, people may have a T or a G), or it could be a large region of DNA (e.g. more than 1 million bases) which is absent in some individuals. The major causes of genetic variation are 1) mutations (changes to the sequence) that occur in the eggs or sperm, and 2) recombination, which is the exchange of DNA between pairs of chromosomes during the formation of sperm and egg cells.

5. How do you measure DNA variants at the molecular level?

There are two main ways to read the information in the DNA: genotyping and sequencing. The difference between them lies in the amount and type of genetic information surveyed. Genotyping looks for specific letters in your genetic sequence which are known to often vary between individuals, whereas sequencing surveys all bases in the order they are in your DNA (including those that may only be variable in you or in a tiny number of people). Most of our study was based on genotyping data - each individual’s DNA was examined on a small slide that allowed us to measure about half a million common genetic variants. We also made use of sequencing data for part of the study, to find rare genetic variants that could cause disease if present

on both chromosomes of an individual, that is on those inherited from both father and mother.

How do you summarise that genetic variation to learn things about human populations?

In humans, genetic variation can be measured by the comparison of one individual against another or against a representative genome sequence of the human species, known as a "reference genome". Genetic differences can also be measured between and within human populations or groups, comparing multiple individuals at the same time. These population-scale comparisons are extremely useful to understand the structure and dynamics of populations. For example, we can ask whether there are subpopulations (e.g. clan groups), whether they are more or less similar to other populations, when they formed, and to what extent people are more likely to choose a partner from the same subpopulation as them. Many of the analyses we do try to address these questions by examining the frequency of different genetic variants in different populations, or the patterns of correlation between different genetic variants scattered around the genome.

What are the major causes of changes in genetic variation?

Genetic variation is generally considered advantageous for a population because it allows some individuals to adapt to new environments and this increases the chance of the survival of the population. However, some genetic variants are disadvantageous because they cause severe diseases or make individuals less likely to have children. There are various factors that can reduce genetic variation in a population over time, including:

- Nonrandom unions: these occur when an individual chooses to have a child with another based on certain characteristics, such as physical traits or social practices. Two special instances of nonrandom unions include consanguineous marriage (marriage between blood relatives e.g. cousins) and endogamy (the practice of marrying within a specific ethnic or social group).
- Genetic drift: this is defined as the random fluctuation of the frequency of variants already present in the population. Strong genetic drift is common when a population experiences a significant reduction in size, called a population bottleneck. A bottleneck might occur for several reasons, for example, a natural disaster which leads to the death of many individuals, a migration event (e.g. a small number of people move to an island and henceforth only have children with people on that island), or a societal change which leads to a large increase in endogamous marriage rates.

6. What did you do in this study?

In this study, we investigated the genetic characteristics and the genetic history of the British Pakistani population. The Pakistani population contains different ethinic groups, such as Punjabis and Kashmiris. Many Pakistanis identify with a lineage-based system of kinship groups, which we refer to here as the biraderi system. Historically, biraderi were endogamous communities; people tended to marry within their own biraderi to reinforce hereditary social status, professional occupation and land ownership. The biraderi system is still important in Pakistan and among British Pakistanis. We explored the impact of the biraderi social system on shaping the genetic variability through marital practices we see today in the British Pakistani population. We also examined the effect of consanguineous marriage (marriage between individuals who are related as second cousins or closer) on patterns of genetic variation in this population. To address these questions, we used the genomes of over 4,000 Pakistani ancestry individuals living in Bradford, UK. These individuals are members of a large cohort study, Born in Bradford, that is investigating the environmental, social and genetic causes of poor health and life outcomes in one of the most deprived cities in the UK, using data from over 10,000 families. Blood or saliva samples were taken from the mothers, their children, and some of the fathers who had volunteered to join this study, and DNA was extracted from these. At the time of recruitment, the mothers were asked, to the best of their knowledge, to report their biraderi group, whether their parents were cousins, and the place they or their parents originate from in Pakistan. This information was really valuable to guide us in the genetic analyses and conclusions of this study.

7. What did you find?

As expected, we found that the Bradford Pakistani groups are genetically similar to other Pakistani and Indian populations. We also found that the biraderi social system played an important role in shaping the genetic variability of the British Pakistani population. We found that the Bradford groups share the same genetic history until around 2,000 years ago when they started separating into the groups we know today. The Pathan are the most distinct group, followed by the Jatt, Choudhry, Bains and a subgroup of the Rajput.

We applied a method to learn about historical effective population size from the genetic data. Effective population size is not the same as the true (census) population size, but rather reflects the proportion of people in the population who are having children, and their marriage patterns; for example, if a country has a total population size of 1 million people, but, for generations, everyone chooses their partner from a small pool of individuals (e.g. the 500 individuals who live in their village), the effective population size will be much smaller than 1 million. Analysis of the Bradford Pakistani groups showed that at various points in history, some groups have seen reductions in effective population size. This could be to do with an increase in endogamy over time making the group more exclusive, or it could be an actual reduction in the population size as recorded in census data. Historical records suggest an increase of endogamous practices in these groups in the last 300-400

years, which may explain the reduction in population size that the genetic data indicates over this time period.

We also developed a new way of working out whether and how an individual's parents were related using his/her genetic data. We compared the findings from this new approach to the self-reported information about parental relatedness. Overall, we found that the self-reported information was somewhat accurate, but some people's parents were more related to each other than was reported, and others' parents were less related than reported. This may be because people are not aware of the extent to which their parents were blood relations, or may not know about relationships between more distant ancestors (e.g. great-great-grandparents), relationships which will alter the effective genetic relationship between their parents.

8. What does the study say about risks of marrying someone from the same biraderi as you? And what about cousin marriage?

Couples who are closely related (e.g. in which the husband and wife are cousins) are at increased risk of having a child with a rare genetic disorder such as severe learning disability. Specifically, they are at higher risk of having a child with a so-called recessive disorder, in which one has to inherit variants that both impact the function of the same gene from both parents to be affected. This risk does not change if the husband or the wife are from their mother or father's sides of the family. Recessive disorders are also more common in the children of couples in which the husband and wife are not closely related but come from the same clan, caste or kinship group (such as a biraderi group), since they are likely to be distantly related. This is particularly the case if people from that group have been marrying within the group for many generations.

We found that for some biraderi groups, marriages between two unrelated people from the same biraderi were more likely to result in a child with a recessive disorder than marriages between two unrelated people from different biraderi groups (notably, the risk was about three times higher for marriages between two Jatts than between one Jatt and someone from another group; it was about six times higher for Bains). However, marriages between cousins carried a much higher risk than marriages between unrelated people from the same biraderi (at least ten times higher for children of first cousins than for children of unrelated parents from the same biraderi, for any of the biraderi groups). For technical reasons, we cannot estimate *absolute* risks of a given type of marriage e.g. what is the overall chance that a child of two unrelated individuals from a given biraderi will have a rare developmental disorder? However, we can estimate *relative* risks of recessive disorders e.g. that a child of first cousins is at least ten times more likely than offspring of unrelated parents from the same biraderi to have a recessive disorder. We would need a different study design to investigate this further.

9. What were some of the limitations of this study?

Our study had some technical limitations including the type of genetic data and methods available to us. We were relying on self-reported information about people's biraderi groups, which may not be accurate. (However, the genetic data suggests that they were at least somewhat accurate for some biraderi groups, since most people who reported coming from that biraderi were more similar to each other genetically than they were to people who reported coming from a different biraderi). Furthermore, our analyses were restricted only to Bradford Pakistanis and these results might not be extendable to the entire Pakistani population. Importantly, there are limitations in our analyses of the risk of diseases due to marriage between relatives or between two people from a given biraderi group. Firstly, we did not have data on actual patients with rare diseases - our findings are simply based on inference from creating hypothetical pairs of parents from amongst healthy individuals, and predicting which would be at risk of having an affected child based on their DNA sequences. Our analysis was also limited to genetic variants which have been previously shown to cause disease when inherited from both mother and father (or seem like they would obviously do so), and ignores other rarer variants which have not been seen before but which may be disease-causing. Our findings would need to be confirmed and extended in cohorts of rare disease patients.

10. Why are these findings important?

This is the first large-scale genetic study of the British Pakistani population, and the first to explore the impact of the biraderi social system on genetic variation. These findings demonstrate the impact of marriage practices and partner choice on population structure and genomic diversity among Pakistanis. This research will guide how medical genetic studies are carried out in this population in the future, ensuring that discoveries we make about links between genetic variants and disease risk are robust. This will ultimately help us to make personalised medicine a reality for Pakistani populations, and teach us valuable lessons that will be useful for other populations too.

11. Are these findings important for other human populations?

There are other populations in the world that, whether due to cultural practices embedded in historical experiences, past population bottlenecks or isolation, might have an increased risk of developing certain disorders. For instance, genetic research has shown that some Indian and Middle Eastern populations might be at increased risk for recessive disorders due to cultural practices such as endogamy and consanguinity. Furthermore, populations that have experienced a reduction in population size, such as the Ashkenazi Jews and Finns, might also have a higher incidence of recessive disorders. Thus, cultural practices as well as many other factors, including in the case of Ashkenazi Jews the experience of persecution, can influence the population structure and the incidence of recessive disorders in human populations.

12. How were the British Pakistanis involved in this study?

The Born in Bradford study has been underway since 2007 and has had considerable input on study design and on how research findings can be used to improve health and wellbeing in the city. This input has included seeking the advice of study participants and sharing findings in many publications and meetings that study members and other local people can access. Specifically for this study we had advice from people of Pakistani heritage living in Bradford who were recognised locally as having a particular interest in and knowledge of their local communities, including the historic and contemporary importance of biraderi groups.

One of the authors of this study (Dr Sufyan Abid Dogra) is an anthropologist from the Bradford Pakistani community. Additionally, we attended the Born in Bradford Annual Conference to discuss this work with interested members of the community, including schoolchildren.

Glossary

- **Genetic population structure** - the organisation of genetic variation within and between populations; for example, are there subpopulations within a country (e.g. clan groups) and are they more or less similar to populations from other countries?
- **Genetic demographic history -** historical characteristics of human populations. In genetic terms, this includes understanding how population sizes or migration rates between populations might have changed over time.
- **Biraderi** - a kinship group with a shared identity (e.g. Jat, or Rajput) within a larger lineage-based system of kinship groups, which we refer to as the biraderi system.
- **DNA** - deoxyribonucleic acid, the molecule carrying genetic instructions for our cells in the form of a string of molecular building blocks called ‘bases’
- **Bases** - small molecules that are joined together in a long string to form DNA; these exist in four types, nicknamed A, C, T and G
- **Chromosomes -** long pieces of DNA. In humans, our genome is made up of 23 pairs of chromosomes, ranging in size from about 46 million to 248 million bases. We inherit one set of 23 chromosomes from each parent.

- **Genetic variant** - a position in the DNA at which more than one form exists in the population; for example, there may be two different bases that can be found at that position, or there may be a large chunk of DNA that is present in some but not all individuals
- **Mutation** - the process by which genetic variants are generated (essentially, errors introduced into DNA by faulty biochemical processes)
- **DNA sequencing** - the process of figuring out the full sequence of an individual's genome
- **Genotyping** - the process of measuring which version of a genetic variant an individual has at a given position in the genome which is known to be variable between people (e.g. does the individual have an A or a C?)
- **Consanguineous marriage** – marriage to someone related genetically, as a second cousin or closer.
- **Endogamy** - the practice of marrying someone from the same specific ethnic or social group (e.g. from the same caste or from the same biraderi).
- **Bottleneck** - a reduction in the effective population size of a population as a result of either a true reduction in the census size (e.g. due to a natural disaster), or a reduction in the size of the pool of individuals from which a given individual chooses a partner (e.g. increased endogamy).
- **Population size** - the number of individuals within a population; in genetics, this term generally refers specifically to '*effective* population size' which reflects not just the number of individuals in the population but also how many of them are having children and contributing to the gene pool of the next generation (e.g. To take an extreme case, there may be 1 million people in a population, but if only 1% of them have children, in genetic terms, the effective population size will be much smaller than 1 million.)
- **Cousin -** two individuals who are related by descent by two or more generations from their most recent common ancestor.
- **Recessive disorder** - a genetic disorder that is caused by having two different damaging genetic variants in the same gene, one inherited from each parent.

References

1. Patterson, N., Price, A. L. & Reich, D. Population structure and eigenanalysis. *PLoS Genet.* **2**, e190 (2006).
2. Alexander, D. H., Novembre, J. & Lange, K. Fast model-based estimation of ancestry in unrelated individuals. *Genome Res.* **19**, 1655–1664 (2009).
3. Narasimhan, V. M. *et al.* The formation of human populations in South and Central Asia. *Science* **365**, (2019).
4. Pathak, A. K. *et al.* The Genetic Ancestry of Modern Indus Valley Populations from Northwest India. *Am. J. Hum. Genet.* **103**, 918–929 (2018).
5. Patterson, N. *et al.* Ancient admixture in human history. *Genetics* **192**, 1065–1093 (2012).
6. Reich, D., Thangaraj, K., Patterson, N., Price, A. L. & Singh, L. Reconstructing Indian population history. *Nature* **461**, 489–494 (2009).
7. Moorjani, P. *et al.* Genetic evidence for recent population mixture in India. *Am. J. Hum. Genet.* **93**, 422–438 (2013).
8. Grugni, V. *et al.* Ancient migratory events in the Middle East: new clues from the Y-chromosome variation of modern Iranians. *PLoS One* **7**, e41252 (2012).
9. Ayub, Q. & Tyler-Smith, C. Genetic variation in South Asia: assessing the influences of geography, language and ethnicity for understanding history and disease risk. *Brief. Funct. Genomic. Proteomic.* **8**, 395–404 (2009).
10. Underhill, P. A. *et al.* Y chromosome sequence variation and the history of human populations. *Nat. Genet.* **26**, 358–361 (2000).
11. Poznik, G. D. *et al.* Punctuated bursts in human male demography inferred from 1,244 worldwide Y-chromosome sequences. *Nat. Genet.* **48**, 593–599 (2016).
12. Freeman, L., Brimacombe, C. S. & Elhaik, E. aYChr-DB: a database of ancient human Y

haplogroups. *NAR Genom Bioinform* **2**, lqaa081 (2020).

13. Rishishwar, L. & Jordan, I. K. Implications of human evolution and admixture for mitochondrial replacement therapy. *BMC Genomics* **18**, 140 (2017).
14. Kivisild, T. *et al.* The genetic heritage of the earliest settlers persists both in Indian tribal and caste populations. *Am. J. Hum. Genet.* **72**, 313–332 (2003).
15. Quintana-Murci, L. *et al.* Where west meets east: the complex mtDNA landscape of the southwest and Central Asian corridor. *Am. J. Hum. Genet.* **74**, 827–845 (2004).
16. Metspalu, M. *et al.* Most of the extant mtDNA boundaries in south and southwest Asia were likely shaped during the initial settlement of Eurasia by anatomically modern humans. *BMC Genet.* **5**, 26 (2004).
17. Chaubey, G. *et al.* Phylogeography of mtDNA haplogroup R7 in the Indian peninsula. *BMC Evol. Biol.* **8**, 227 (2008).
18. Dai, C. L. *et al.* Population histories of the United States revealed through fine-scale migration and haplotype analysis. doi:10.1101/577411.
19. GenomeAsia100K Consortium. The GenomeAsia 100K Project enables genetic discoveries across Asia. *Nature* **576**, 106–111 (2019).
20. Nakatsuka, N. *et al.* The promise of discovering population-specific disease-associated genes in South Asia. *Nat. Genet.* **49**, 1403–1407 (2017).
21. Mezzavilla, M. Neon: An R Package to Estimate Human Effective Population Size and Divergence Time from Patterns of Linkage Disequilibrium between SNPS. *Journal of Computer Science & Systems Biology* vol. 8 (2015).
22. Peng, B. & Kimmel, M. simuPOP: a forward-time population genetics simulation environment. *Bioinformatics* **21**, 3686–3687 (2005).
23. McEvoy, B. P., Powell, J. E., Goddard, M. E. & Visscher, P. M. Human population dispersal 'Out of Africa' estimated from linkage disequilibrium and allele frequencies of SNPs. *Genome Res.* **21**, 821–829 (2011).
24. Bhérer, C., Campbell, C. L. & Auton, A. Refined genetic maps reveal sexual dimorphism in human meiotic recombination at multiple scales. *Nat. Commun.* **8**, 14994 (2017).

25. Narasimhan, V. M. *et al.* Estimating the human mutation rate from autozygous segments reveals population differences in human mutational processes. *Nature Communications* vol. 8 (2017).
26. Garson, D. G. Interpreting neural network connection weights. (1991).
27. Severson, A. L., Carmi, S. & Rosenberg, N. A. Variance and limiting distribution of coalescence times in a diploid model of a consanguineous population. *bioRxiv* 2020.06.30.180521 (2020) doi:10.1101/2020.06.30.180521.
