# Supplementary material for: Fine-scale population structure and demographic history of British Pakistanis
Source: Nat Commun. 2021 Dec 10;12:7189. doi: 10.1038/s41467-021-27394-2 (PMC8664933; doi:10.1038/s41467-021-27394-2)
Supplement: Supplementary file 21 — Reporting Summary [file 41467_2021_27394_MOESM21_ESM.pdf]

nature research

Corresponding author(s): Dr Hilary Martin

Last updated by author(s): Oct 18, 2021

Reporting Summary

Nature Research wishes to improve the reproducibility of the work that we publish. This form provides structure for consistency and transparency in reporting. For further information on Nature Research policies, see our Editorial Policies and the Editorial Policy Checklist.

Statistics

For all statistical analyses, confirm that the following items are present in the figure legend, table legend, main text, or Methods section.

n/a	Confirmed	
☐	☒	The exact sample size (*n*) for each experimental group/condition, given as a discrete number and unit of measurement
☐	☒	A statement on whether measurements were taken from distinct samples or whether the same sample was measured repeatedly
☐	☒	The statistical test(s) used AND whether they are one- or two-sided<br>*Only common tests should be described solely by name; describe more complex techniques in the Methods section.*
☐	☒	A description of all covariates tested
☐	☒	A description of any assumptions or corrections, such as tests of normality and adjustment for multiple comparisons
☐	☒	A full description of the statistical parameters including central tendency (e.g. means) or other basic estimates (e.g. regression coefficient) AND variation (e.g. standard deviation) or associated estimates of uncertainty (e.g. confidence intervals)
☐	☒	For null hypothesis testing, the test statistic (e.g. *F*, *t*, *r*) with confidence intervals, effect sizes, degrees of freedom and *P* value noted<br>*Give P values as exact values whenever suitable.*
☐	☒	For Bayesian analysis, information on the choice of priors and Markov chain Monte Carlo settings
☒	☐	For hierarchical and complex designs, identification of the appropriate level for tests and full reporting of outcomes
☐	☒	Estimates of effect sizes (e.g. Cohen's *d*, Pearson's *r*), indicating how they were calculated

Our web collection on statistics for biologists contains articles on many of the points above.

Software and code

Policy information about availability of computer code

Data collection	No software was used
Data analysis	The code for inferring parental relatedness is available at https://github.com/malawsky/consanguinity_simulation.<br>The code implementing the model of IBD and ROH distributions can be accessed at https://github.com/scarmi/ibd_roh.<br>The code for the IBD scores was provided by Nathan Nakatsuka and David Reich and will be made available upon request to them.<br>PLINK v1.90b4,EIGENSOFT 7.2.1,KING 2.2.4,ADMIXTURE v1.3,ADMIXTOOLS v6.0,fineSTRUCTURE v4.0.1,SHAPEIT v2.12,PopART v1.7,<br>IBDseq v.r1206,GERMLINE 1.5.3,Eagle v2.4.1,IBDNe v.19Sep19.268,bcftools/roh 1.13,GARLIC v1.6.0a,simuPOP v.1.9,Haplogrep2,yHaplo,4P<br>R packages: uwot, Ade4, NeON, nnet

For manuscripts utilizing custom algorithms or software that are central to the research but not yet described in published literature, software must be made available to editors and reviewers. We strongly encourage code deposition in a community repository (e.g. GitHub). See the Nature Research guidelines for submitting code & software for further information.

Data

Policy information about availability of data

All manuscripts must include a data availability statement. This statement should provide the following information, where applicable:

- Accession codes, unique identifiers, or web links for publicly available datasets
- A list of figures that have associated raw data
- A description of any restrictions on data availability

The genetic data and questionnaire data (covering self-reported consanguinity, village of origin and biraderi group) from Born in Bradford can be obtained as described here https://borninbradford.nhs.uk/research/how-to-access-data/ .

Field-specific reporting

Please select the one below that is the best fit for your research. If you are not sure, read the appropriate sections before making your selection.

☒ Life sciences ☐ Behavioural & social sciences ☐ Ecological, evolutionary & environmental sciences

For a reference copy of the document with all sections, see nature.com/documents/nr-reporting-summary-flat.pdf

Life sciences study design

All studies must disclose on these points even when the disclosure is negative.

Sample size	No methods were used to predetermine sample size. The sample size was determined by the number of samples recruited by the BiB project, which was limited by funding, the number of women fulfilling the recruitment criteria, and the success rate of recruitment.
Data exclusions	Some samples and SNPs were excluded as described in the Methods under the sections "Quality control of genotype chip data" and "Inference and removal of relatives". We have restricted to using methods appropriate for this sample size and given confidence intervals where appropriate to reflect the uncertainty inherent in the limited sample size.
Replication	No replication sample was available.
Randomization	Randomization was not applicable because this was a descriptive population genetic study rather than one involving experiments.
Blinding	Blinding was not applicable because this was a descriptive population genetic study rather than one involving experiments.

Reporting for specific materials, systems and methods

We require information from authors about some types of materials, experimental systems and methods used in many studies. Here, indicate whether each material, system or method listed is relevant to your study. If you are not sure if a list item applies to your research, read the appropriate section before selecting a response.

Materials & experimental systems

n/a	Involved in the study	
☒	☐	Antibodies
☒	☐	Eukaryotic cell lines
☒	☐	Palaeontology and archaeology
☒	☐	Animals and other organisms
☐	☒	Human research participants
☒	☐	Clinical data
☒	☐	Dual use research of concern

Methods

n/a	Involved in the study	
☒	☐	ChIP-seq
☒	☐	Flow cytometry
☒	☐	MRI-based neuroimaging

Human research participants

Policy information about studies involving human research participants

Population characteristics	Just under half the participants of the Born in Bradford study are of Pakistani origin, the remainder mainly white European. The mothers were of reproductive age at the time of sampling (mostly between 20 and 40 years old).
Recruitment	The Born in Bradford cohort study was established in 2007 to examine how genetic, nutritional, environmental, behavioural and social factors impact on health and development during childhood, and subsequently adult life in a deprived multi-ethnic population. Between 2007 and 2011, detailed information on socio-economic characteristics, ethnicity and family trees, lifestyle factors, environmental risk factors and physical and mental health was collected from 12,453 women with 13,776 pregnancies and 3,448 of their partners. It's possible people from particular biraderi group or people who were/were not consanguineous were more likely to take part, and this may have impacted results. However, a high fraction (>80%, https://doi.org/10.1093/ije/dys112) of those invited to join did so, so this seems unlikely.
Ethics oversight	Ethical approval for the data collection was granted by Bradford Research Ethics Committee (Ref 07/H1302/112).

Note that full information on the approval of the study protocol must also be provided in the manuscript.
